# PMEPA1 promotes gastric cancer cell proliferation by regulating the ubiquitin-mediated degradation of 14-3-3σ and promoting cell cycle progression

**DOI:** 10.1590/1414-431X2024e13985

**Published:** 2024-11-25

**Authors:** Heyuan Huang, Ruizheng Sun, Yi Xu, Renchao Liu, Zihua Chen

**Affiliations:** 1 The Hunan Provincial Key Lab of Precision Diagnosis and Treatment for Gastrointestinal Tumor, Xiangya Hospital, Central South University, Changsha, Hunan, China; 2 Department of General Surgery, Xiangya Hospital Central South University, Changsha, Hunan Province, China; 3 National Clinical Research Center for Geriatric Disorders, Xiangya Hospital, Central South University, Changsha, Hunan, China

**Keywords:** PMEPA1, Cell cycle, 14-3-3σ, TTC3, Gastric cancer

## Abstract

Gastric cancer (GC) remains a global health challenge due to its heterogeneity and diverse regional epidemiology. Treatment for advanced GC often requires chemotherapy, whose effects are closely associated with the cell cycle. This association highlights the critical need to understand cell cycle regulators that can influence the effectiveness of chemotherapy. Bioinformatics analyses were performed on transcriptome data from a hospital cohort and on a publicly available database. Flow cytometry was used for cell cycle analysis. The interaction of PMEPA1 with 14-3-3σ was confirmed by coimmunoprecipitation and immunofluorescence staining. Western blot analysis was performed following inhibition of protein synthesis and degradation to assess 14-3-3σ protein stability, while ubiquitination was evaluated after treatment with the proteasome inhibitor MG132. High *PMEPA1* expression was detected in GC tissues and was correlated with poor prognosis. *In vitro* overexpression of *PMEPA1* promoted GC cell proliferation, while knockdown of *PMEPA1* inhibited cell proliferation and induced G2/M arrest. *In vivo* study showed that overexpressing *PMEPA1* promoted tumor growth, while knocking down *PMEPA1* inhibited tumor growth, as indicated by the level of the proliferation marker Ki67. 14-3-3σ was identified as a downstream target of PMEPA1. PMEPA1 binds to 14-3-3σ and promoted its degradation by facilitating its ubiquitination. Overexpression of PMEPA1 increased its interactions with TTC3 and 14-3-3σ, increased 14-3-3σ ubiquitination, and reduced 14-3-3σ stability, and the opposite effects were observed after PMEPA1 knockdown. PMEPA1 recruited TTC3, allowing the ubiquitination of 14-3-3σ and leading to its degradation, thus promoting cell cycle progression in GC.

## Introduction

Gastric cancer is the fifth most commonly diagnosed cancer and the fourth most common cause of cancer-related death worldwide (1). Despite the advances in understanding the molecular mechanisms of gastric cancer, the efficacy of its treatment is notably hindered by challenges in early detection and the complex tumor microenvironment. Therefore, there is a need to further explore the pathogenic mechanisms of and treatment methods for gastric cancer.

To that end, our team collected gastric cancer and adjacent non-tumor specimens and submitted two pairs of specimens for transcriptome sequencing. Differential expression analysis revealed *PMEPA1* overexpression in cancer specimens (Supplementary Figure S1A and B), which was subsequently confirmed in pathological samples. Prostate transmembrane protein androgen induced 1 (PMEPA1) is classified as a type 1b transmembrane protein with luminal, membrane spanning and cytoplasmic domains (2). The role of *PMEPA1* differs across tumors. Studies have shown that *PMEPA1* is an overexpressed oncogene in solid tumors such as renal, breast, and cervical cancers (3,4). *PMEPA1* was found to promote the progression of pancreatic cancer by activating the MAPK signaling pathway (5). *PMEPA1* was also shown to activate EMT and accelerate the proliferation and metastasis of colorectal cancer cells (6).

However, *PMEPA1* may also play a tumor-suppressive role. Some researchers have reported that silencing *PMEPA1* can activate the androgen pathway, thereby accelerating the progression of prostate cancer (7,8). The interaction between PMEPA1 and the E3 ubiquitin ligase NEDD4 in prostate cancer cells may play a role in suppressing tumor cell activity (9,10). *PMEPA1* specifically inhibits AR signal transduction, thereby suppressing the proliferation and colony formation of androgen-responsive prostate cancer cells (11). Studies have reported that *PMEPA1* plays a role in promoting tumor development in gastrointestinal tumors (12,13). Although *PMEPA1* has been previously reported to promote the development of various tumors, its effects on the growth of gastric cancer, especially its influence on the cell cycle, remain unclear.

The term “cell cycle” refers to the series of intracellular events that lead to cell division and replication (14). Aberrant cell cycle progression is one of the fundamental mechanisms underlying tumorigenesis; thus, regulators of the cell cycle machinery are rational anticancer therapeutic targets (15). In the literature, it has been reported that cell cycle regulators are related to the prognosis of GC patients (16). Targeting cell cycle-associated proteins, including cyclin-dependent kinases and checkpoint kinases, has gradually become a promising molecular therapeutic strategy (17).

Therefore, research on the molecular mechanisms involved in the cell cycle has become particularly important for improving the treatment of gastric cancer. Our mass spectrometry analysis revealed that PMEPA1 can bind to key cell cycle checkpoint proteins. Hence, we focused on the cell cycle and tumor growth, investigating the underlying mechanisms of *PMEPA1*, potentially identifying it as a novel therapeutic target.

## Material and Methods

### Public database analysis

The gene expression matrix and annotation information for stomach adenocarcinoma were obtained from The Cancer Genome Atlas (TCGA, https://www.cancer.gov/tcga/). The dataset contained data for 448 stomach adenocarcinoma (STAD) cases, namely 36 normal specimens and 412 tumor specimens. Additionally, gene expression matrices, sample information, and survival data related to Gene Expression Omnibus (GEO) datasets were obtained from the GEO database (http://www.ncbi.nlm.nih.gov/geo/). Specifically, the GSE26899 (18) dataset contains 108 patient specimens, which include 12 normal tissue samples and 96 tumor tissue samples. The GSE84437 (19) dataset contains gene expression data for 483 tumor patients, along with their clinical information. The TCGA-STAD, GSE26899, and GSE84437 datasets were used to analyze the expression level of *PMEPA1* in gastric cancer tissues and normal tissues. Survival curves were generated using the R software (version 4.1.2; R Core Team) packages “survival” and “survminer”.

### Patient collection and selection

The patients in the Xiangya cohort used in this study were selected from the Department of Gastrointestinal Surgery at Xiangya Hospital, Central South University, between 2017 and 2020. The selection criteria for the collection of clinicopathological data included a diagnosis of stomach adenocarcinoma, treatment with surgery with or without adjuvant radiochemotherapy, a T stage of T1 to T4a, and no imaging evidence of distant metastasis to organs such as the liver or lungs. A total of 166 patients met these criteria. Subsequently, we conducted telephone follow-ups with these 166 patients; 106 patients were successfully contacted, while 60 were lost to follow-up. We compiled survival status data for the 106 patients contacted and grouped the patients according to the staining scores of PMEPA1 markers in pathological sections. Survival curves were plotted based on these data. Pathological slides were obtained from the Pathology Department of Xiangya Hospital. Specimen collection and usage were conducted with informed consent from the patients.

### Immunohistochemistry and scoring of staining

Paraffin sections were subjected to deparaffinization, rehydration, and antigen retrieval. Endogenous peroxidase was inactivated with hydrogen peroxide. The sections were blocked and incubated with primary antibodies against PMEPA1 (1:200, 16521-1-AP, Proteintech, China) and Ki67 (1:2000, 27309-1-AP, Proteintech) overnight at 4°C. After incubation with a secondary antibody (goat anti-rabbit IgG H&L, 1:500, #ab6702, Abcam, USA), DAB and hematoxylin were applied. Staining was scored based on the percentage of positive cells and the intensity of staining.

### Cell culture

The human gastric cancer cell lines MKN-7 (#CL-0574, Procell, China), BGC-823 (#CL-0033, Procell), HGC-27 (#CL-0107, Procell), and MKN-45 (#CL-0292, Procell) and the human gastric epithelial cell line GES-1 (#CL-0563, Procell) were cultured in complete RPMI 1640 medium (Procell) supplemented with 10% fetal bovine serum (FBS; Gibco, USA). The AGS cell line (#CL-0022, Procell) was cultured in Ham's F-12 complete medium (Procell) supplemented with 10% FBS. The HEK-293T cell line (# CL-0005, Procell) was cultured in high-glucose complete DMEM (Procell) supplemented with 10% FBS. All complete culture media were supplemented with 1% 100 μg/mL penicillin-streptomycin. The cells were maintained in a humidified CO_2_ incubator at 37°C.

### Western blotting

SDS-PAGE was performed using 5% stacking and 10% resolving gels. Proteins were separated by electrophoresis and transferred to a PVDF membrane. The membrane was blocked with 5% skim milk and incubated overnight with primary antibodies at 4°C. After washing, the membrane was incubated with secondary antibodies (goat anti-rabbit IgG, 1:2000, #SA00001-2, Proteintech), washed again, and visualized using an ECL system. Primary antibodies specific for the following proteins were used: 14-3-3σ (1:50, #ET1612-41, HuaBio, USA), PMEPA1 (1:500, #16521-1-AP, Proteintech), TTC3 (1:2000, #ab80061, Abcam), Cdc25c (1:500, #ER1706-38, HuaBio), p-Cdc25c (ser216, 1:500, #bs-3096R, Bioss, USA), cdk1 (1:1000, #10762-1-AP, Proteintech), cyclinB1 (1:1000, #28603-1-AP, Proteintech), p-cdk1 (Tyr15, 1:500, #44-686G, Invitrogen, USA), Ubiquitin (1:1000, #3933, Cell Signaling Technology, USA), α-tubulin (1:5000, #11224-1-AP, Proteintech), HA (1:1000, #3724, Cell Signaling Technology), Flag (1:1000, #14793, Cell Signaling Technology), and α-actin (1:3000, #23660-1-AP, Proteintech).

### Cell viability assay

Cells in the logarithmic growth phase were harvested, counted, and seeded onto a 96-well plate at 1000 cells/well. Cell proliferation was assessed on days 1, 2, 4, and 5 using the 3-(4,5-dimethylthiazol-2yl)-2,5-diphenyltetrazolium bromide (MTT) assay. After the addition of 20 μL of 5 mg/mL MTT, the cells were incubated for 4 h; DMSO was then added, and the mixture was incubated with shaking for 10 min. The absorbance at 570 and 630 nm was measured. Cell proliferation was calculated by subtracting the absorbance at 630 nm from the absorbance at 570 nm.

### Plasmid construction and generation of stable cell lines

For *PMEPA1* and *14-3-3σ* overexpression, HA-tagged primers (for *PMEPA1*) and FLAG-tagged primers (for *14-3-3σ*) (Supplementary Table S1) were used to amplify the corresponding CDS from MKN-7 cell cDNA, and the sequences were subsequently inserted into the plv19 vector, with an empty vector serving as a control. The overexpression plasmid (pCMV3) containing the full-length cDNA ORF of *TTC3* was obtained from Sino Biological, Inc. (#HG18115-CH, China).

The *PMEPA1* and *14-3-3σ* knockdown plasmids were constructed by inserting the annealed shRNA primers into the plko.1 vector (shRNA primers for target genes, see Supplementary Table S1). A scrambled shRNA was used as a control.

siRNAs targeting specific initiation sites, acquired from Sigma-Aldrich (#EHU078191), were used to knock down the expression of *TTC3*.

### Generation of stable cell lines and transient transfection

After vector construction, we packaged the sh-*PMEPA1* vector, sh-14-3-3σ vector, scramble vector, OE-*PMEPA1* vector, and OE-14-3-3σ vector separately into lentiviral particles for cell infection, generated stable cell lines through antibiotic selection as previously described, and confirmed successful transduction by western blotting. siRNAs and the *TTC3* overexpression plasmid were diluted in serum-free medium, mixed with Lipofectamine 2000, and added to the cell cultures at final concentrations of 50 nmol/L (siRNAs) and 2 µg/mL (plasmid). Six hours later, the culture medium was replaced with fresh complete culture medium. Forty-eight hours after transfection, cells were harvested for further experiments.

### Transcriptome sequencing

First, the samples were subjected to meticulous testing to ensure their quality, and mRNA isolation was then performed using oligo (dT) magnetic beads. The subsequent steps involved fragmenting the isolated mRNA and synthesizing single- and double-stranded cDNA. The double-stranded cDNA was subjected to end repair, adenylation, and adapter ligation prior to PCR amplification of the corresponding products. Library testing was performed for quality control, and the PCR products were then circularized and linear DNA molecules were digested. Finally, single-stranded circular DNA molecules were sequenced and converted into DNA nanoballs (DNBs), which were subjected to sequencing using high-density DNA nanochips and combinatorial probe-anchor synthesis (cPAS) technology. The thresholds for selecting the differentially expressed genes shown in the volcano plot were calculated as follows: analysis between gastric cancer tissues and adjacent non-tumor tissues (Supplementary Figure S1B): |log_2_FC| >2 and P-value<0.01; analysis between the *PMEPA1* knockdown group and the control group (Figure 4A): |log_2_FC|>1 and P-value<0.05. Gene enrichment analysis was performed using the R package “clusterProfiler”. Visualization of the enrichment analysis results was achieved using the R packages “enrichplot” and “GOplot”. A Venn diagram was generated using the R package “ggVennDiagram”.

### Immunoprecipitation (IP) and co-IP

Cells were lysed with IP lysis buffer to extract total protein. Approximately 1.2 mg of protein was incubated with a targeted antibody (or control IgG) overnight at 4°C. Protein A/G PLUS agarose beads were added, and the mixture was incubated for 2-3 h and centrifuged (3000 *g*, 4°C, 5 min). The pellet was collected, washed, resuspended in 2× SDS buffer, and then heated at 95°C for 5 min. After these steps, the sample was ready for mass spectrometry or western blot analysis to identify or validate protein interactions. Antibodies specific for PMEPA1 (1-2 µg/mL, #sc-293372, Santa Cruz Biotechnology, USA), 14-3-3sigma (2 µg/mL, #1433S01, Invitrogen), HA (0.5-2 µg/mL, #ab49969, Abcam), and Flag (2 µg/mL, #8146, Cell Signaling Technology), as well as IgG (2.5 mg/mL, #5873, Cell Signaling Technology) were used.

### Immunofluorescence staining

Cells were fixed with 4% formaldehyde solution and then treated with a permeabilizing agent (0.1% Triton X-100) to increase membrane permeability. Next, blocking solution (PBS containing 1% BSA) was used to block nonspecific binding sites and reduce background staining. The cells were then incubated with primary antibodies against PMEPA1 [(1:100, #PA5-49867, Invitrogen), 14-3-3σ (1:100, #MA5-11663, Invitrogen), and TTC3 (1:100, #ab80061, Abcam)] overnight at 4°C. Afterward, the cells were incubated with fluorescently labeled secondary antibodies [Cy3-conjugated goat anti-rabbit IgG H&L (1:200, # A0516, Beyotime, China) and FITC-conjugated goat anti-mouse IgG H&L (1:200, #A0568, Beyotime)] for 1 h at room temperature in the dark. Finally, cell nuclei were stained with the fluorescent dye DAPI and were observed and analyzed by fluorescence microscopy.

### Mass spectrometry

After immunoprecipitation with an anti-HA antibody in AGS cells from the control and overexpression groups, bands were excised from the gel for protein identification by mass spectrometry. In brief, total protein in the gel was extracted and digested with trypsin overnight. Following centrifugation, the digested peptide mixture was extracted and desalted using StageTips (Thermo Fisher Scientific, USA). Then, the purified peptides were dissolved, separated using the EASY-nLC 1200 system (Thermo Fisher Scientific), and analyzed by mass spectrometry on an Orbitrap Exploris™ 480 system (Thermo Fisher Scientific).

### Colony formation assay

Logarithmic-phase gastric cancer cells were detached and collected using trypsin followed by gentle pipetting to obtain a single-cell suspension. The cells were seeded onto a 6-well plate, typically at a density of 200-500 cells per well, and cultured for 2-3 weeks until the appearance of visible colonies. Then, the cells were washed with PBS, fixed with pure methanol, and stained with 0.5% crystal violet staining solution.

### Wound healing assay

Logarithmic-phase gastric cancer cells were grouped, counted, and seeded onto a 12-well plate at a density of 5×10^5^ cells per well for overnight incubation (37°C) to form a monolayer. Next, wounds were made in the plate by scratching the cell monolayer surface using a 10-μL pipette tip. After three washes with PBS, serum-free culture medium was added, and the cells were cultured at 37°C in an incubator for 24 h. Images were acquired under a microscope at the specified time points before and after incubation and saved for further analysis.

### Flow cytometry

MKN-7 cells were synchronized for 24 h in serum-free culture medium before being fixed in 70% ethanol overnight. The ethanol was then removed, and the cells were resuspended in 500 µL of PI staining solution (containing RNase) and then incubated in the dark at 37°C for 30 min. Following incubation, the cells were washed with PBS solution and then resuspended. Cells (1×10^4^) were subjected to flow cytometry to evaluate the cell cycle distribution. The obtained data were analyzed using FlowJo 10 software.

### Transwell assay

Logarithmic-phase gastric cancer cells were counted and seeded onto the membranes of Transwell inserts at a density of 5×10^5^ cells/mL in a volume of 200 µL, 20% FBS medium was added to the lower chamber, and the plate was incubated at 37°C for 48 h. After incubation, the cells were washed and fixed with methanol, and the non-migrated cells were removed. The remaining cells were stained with 0.1% crystal violet for 30 min, rinsed, and imaged. The cells that migrated through the membrane were then counted.

### Real-time quantitative PCR (qRT-PCR) assay

Treated AGS and MKN-7 cells were harvested, and total RNA was extracted with TRIzol (Invitrogen). A reverse transcription kit (TaKaRa, Japan) was used according to the manufacturer's protocol to synthesize cDNA from RNA by reverse transcription. A LightCycler 480 (Roche Diagnostics, USA) fluorescence quantitative PCR instrument was used to assess gene expression, and a fluorescence quantitative PCR kit (SYBR Green Mix, Roche Diagnostics) was used to prepare the reactions according to the manufacturer's protocol. The PCR thermal cycling conditions were as follows: initial denaturation at 94°C for 2-5 min, followed by 35-40 cycles of denaturation at 94°C for 30 s, annealing at 50-60°C for 30 s, and elongation at 72°C for 10 s at 1-2 kb/min. The final amplification cycle was followed by an extension step at 72°C for 5-10 min. The melt curve temperatures ranged from 62-95°C. Each reaction was carried out in triplicate. GAPDH was used for normalization to calculate the relative expression levels of target genes using the 2^-ΔΔCt^ method, where ΔΔCt = experimental group (Ct target gene - Ct internal reference) - control group (Ct target gene - Ct internal reference). The sequences of the primers used for amplification of 14-3-3σ (SFN) were as follows: forward, 5′-CATCATTGACTCAGCCCGGT-3′; reverse, 5′-TGTTGGCGATCTCGTAGTGG-3′.

### Animal experiments

Male NOD-SCID mice (4-6 weeks old) were grouped and injected subcutaneously with a cell suspension (*PMEPA1*-overexpressing AGS cells, *PMEPA1* knockdown MKN-7 cells, and control cells) mixed with a 1:1 Matrigel-PBS solution. Mouse weight and tumor size were monitored every 3-5 days. After the diameter of tumors had grown to 1.5 to 2.0 cm, the mice were euthanized. The tumors were measured, with a portion of the tissue cryopreserved for protein extraction and the remaining tissue fixed with 4% paraformaldehyde for tissue sectioning. The animal experiment was verified and ethically approved by the Medical Ethics Committee of Xiangya Hospital, Central South University.

### Statistical analysis

Statistical analyses were performed using SPSS 26.0 (IBM, USA) and GraphPad Prism 8 software (USA). Quantitative data are reported as means±SD. Comparisons between two groups were conducted using a *t*-test. Correlation analysis between PMEPA1 expression, as determined by the IHC scores, and patient clinicopathological features was conducted using the chi-squared test. The Wilcoxon rank-sum test was used for the unpaired TCGA and GSE cohorts, while the paired Wilcoxon test was applied to the paired TCGA cohort. The Kaplan-Meier method was employed to assess the relationships between gene expression patterns and patient survival prognosis and differences in overall long-term survival and disease-free survival in the TCGA cohort, as well as overall survival differences in the GSE cohort, were assessed using partial and regular log-rank tests, respectively. A significance level of P<0.05 indicated statistical significance.

## Results

### *PMEPA1* was highly expressed in gastric cancer, and high *PMEPA1* expression was associated with poor prognosis in patients with gastric carcinoma

**Figure 1:**
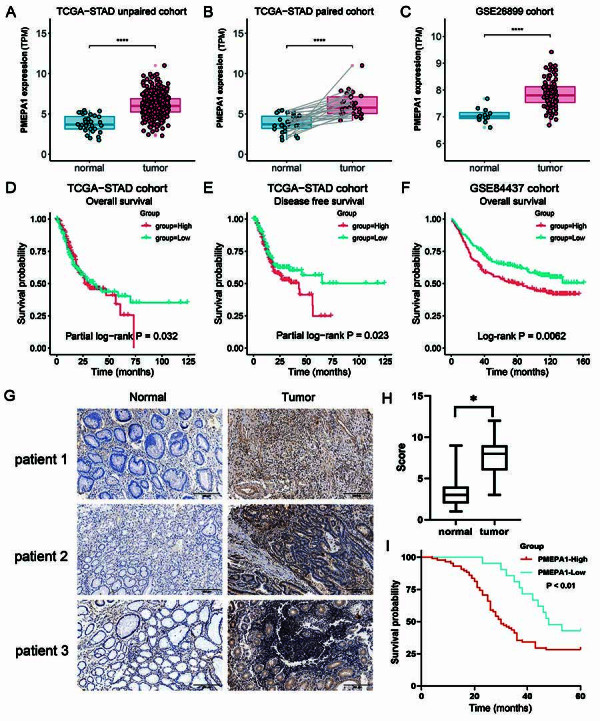
Expression patterns and prognostic values of PMEPA1 both in public databases (TCGA and GEO) and Xiangya cohort. PMEPA1 expression was analyzed in unpaired (**A**) and paired (**B**) TCGA-STAD cohorts, and the GSE26899 cohort (**C**). Kaplan-Meier plots for overall survival (OS) and disease-free survival in the TCGA-STAD cohort (**D**, **E**), OS in the GSE84437 (**F**), and Xiangya Hospital cohorts (**I**) were grouped by PMEPA1 expression. PMEPA1 expression comparison between cancer and adjacent normal tissues (**G,** scale bar 200 μm) and its relationship with pathological staging and scoring in the Xiangya cohort (166 patients) (**H,** data are reported as median and interquartile range) were also examined. Wilcoxon rank-sum test was used for P-values in **A** and **C**, paired Wilcoxon test in **B**, and log-rank test in **I** (*P<0.05, ****P<0.0001). Long-term survival differences in **D** and **E**, and OS differences in **F**, were assessed using partial and regular log-rank tests, respectively.

In the TCGA database, the expression level of *PMEPA1* was elevated in gastric cancer tissues compared with normal tissues in both the paired and unpaired cohorts (Figure 1A and B). Similarly, in the GSE26899 dataset, *PMEPA1* expression was higher in tumor tissues than in normal tissues (Figure 1C). The Kaplan-Meier curve revealed significant differences between the groups with high and low *PMEPA1* expression in overall survival (more than 50 months) (Figure 1D) and progression-free survival (more than 25 months) (Figure 1E). Analysis of the GSE84437 dataset showed that patients with high *PMEPA1* expression had poorer overall survival prognoses than those with low *PMEPA1* expression (Figure 1F).

Immunohistochemical staining was subsequently performed on collected pathological slides, revealing greater PMEPA1 expression in tumor tissues than in adjacent normal tissues (Figure 1G) and significantly higher PMEPA1 staining scores in tumor tissues (Figure 1H). The corresponding patients were categorized into the high-expression (staining score ≥6) and low-expression (staining score <6) groups. A statistical analysis of the clinicopathological information of the 166 eligible patients was conducted. The chi-squared test revealed that high PMEPA1 expression was significantly associated with a high tumor invasion depth, lymph node metastasis, vascular and neural invasion, a high TNM stage, and Ki67 positivity (P<0.05) but not with age, sex, or histological type (P>0.05) (Table 1). By analyzing the survival information of the 106 patients successfully contacted through telephone follow-up, we identified 33 survivors, resulting in a 31.3% (33/106) overall survival rate, with 9 survivors in the high-expression group and 24 in the low-expression group. Patients with high PMEPA1 expression thus had poorer outcomes (Figure 1I).

**Table 1:** Statistical analysis of the correlation between PMEPA1 and clinicopathological data.

Parameters	Cases (166)	PMEPA1		P-value
		Low expression(59; 36%)	High expression(107; 64%)	
Age (years)				
≤50	65 (39%)	27 (16%)	38 (23%)	0.195
>50	101 (61%)	32 (19%)	69 (42%)	
Gender				
Male	98 (59%)	38 (23%)	60 (36%)	0.296
Female	68 (41%)	21 (13%)	47 (28%)	
Primary Tumor (T)				
T1-2	74 (45%)	39 (23%)	35 (22%)	<0.001
T3-4	92 (55%)	20 (13%)	72 (42%)	
Lymph node metastasis				
Yes	89 (54%)	25 (15%)	64 (39%)	0.031
No	77 (46%)	34 (20%)	43 (26%)	
Vascular invasion				
Yes	94 (57%)	21 (13%)	73 (44%)	<0.001
No	72 (43%)	38 (23%)	34 (20%)	
Neural invasion				
Yes	85 (51%)	22 (13%)	63 (38%)	0.008
No	81 (49%)	37 (22%)	44 (27%)	
TNM staging				
I-II stage	37 (22%)	23 (14%)	14 (8%)	<0.001
III-IV stage	129 (78%)	36 (22%)	93 (56%)	
Histological type				
Poor or undifferentiation	86 (52%)	28 (17%)	58 (35%)	0.405
High or moderate differentiation	80 (48%)	31 (19%)	49 (29%)	
Ki67				
≤50%	63 (38%)	36 (22%)	27 (16%)	<0.001
>50%	103 (62%)	23 (14%)	80 (48%)	

Data are reported as number and percentage. P-value <0.05 was considered statistically significant (chi-squared test).

### *PMEPA1* promoted the proliferation and metastasis of gastric cancer cells

**Figure 2:**
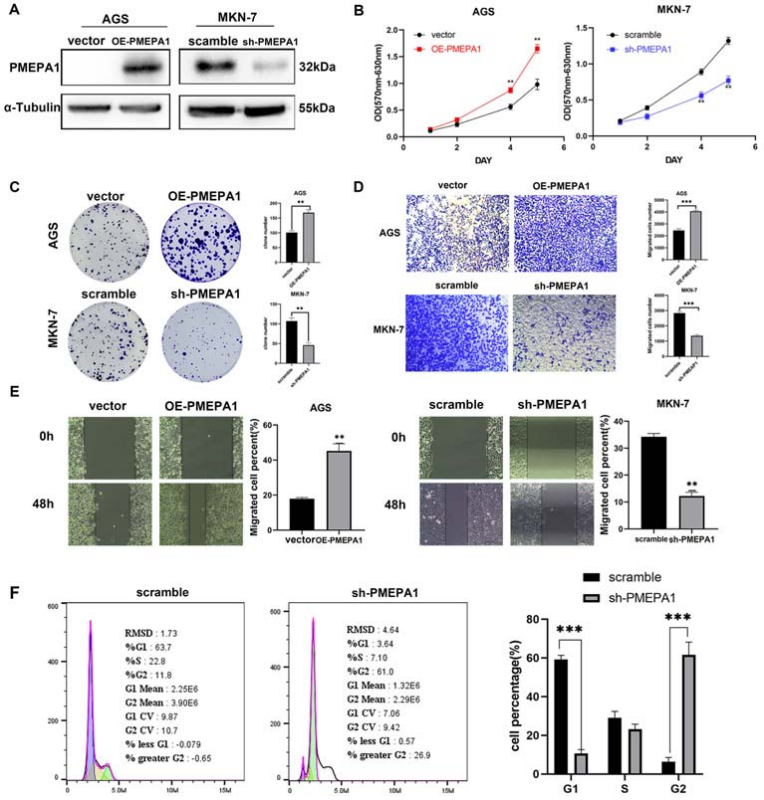
*In vitro* cellular functional experiments of PMEPA1 overexpression and knockdown. Stable cell lines overexpressing PMEPA1 in AGS (OE-PMEPA1) and control (vector), as well as PMEPA1 knockdown (sh-PMEPA1) and control (scramble) in MKN-7, were established and then validated by western blot (**A**). MTT assay was applied to detect cell viability of stably transfected AGS and MKN-7 cells (**B**). Clone formation assay was used to determine cell proliferation of stably transfected AGS and MKN-7 cells (**C**). Transwell assay and wound healing assay were applied to detect cell migration of stably transfected AGS and MKN-7 cells (**D** and **E**). Flow cytometry was used to ascertain cell cycle of stably transfected MKN-7 cells (**F**). Data are reported as means±SD. **P<0.01, ***P<0.001 compared to vector or scramble group; Student's *t*-test.

To explore the role of *PMEPA1* in gastric cancer, *PMEPA1* expression was first assessed in various gastric cancer cell lines, and higher levels were measured in MKN-7 cells, while lower levels were measured in AGS cells (Supplementary Figure S2). Hence, AGS and MKN-7 cells were chosen for *in vitro* experiments. *PMEPA1*-overexpressing AGS cells and *PMEPA1*-silenced MKN-7 cells were generated using an overexpression plasmid and a shRNA plasmid, respectively, after which the transfection efficiency was verified (Figure 2A). The overexpression of *PMEPA1* significantly increased AGS cell viability (Figure 2B), proliferation (Figure 2C), and migration (Figure 2C and E). However, *PMEPA1* knockdown in MKN-7 cells led to reduced cell viability, proliferation, and migration (Figure 2B-E). Notably, *PMEPA1* knockdown induced G2/M arrest in MKN-7 cells, with the percentage of MKN-7 cells in the G2 phase increasing to 61.0% compared to that of control cells (11.8%), explaining the reduction in the proliferation rate following *PMEPA1* knockdown in MKN-7 cells (Figure 2F).

### Knockdown of *PMEPA1* inhibited gastric tumor growth *in vivo*

**Figure 3:**
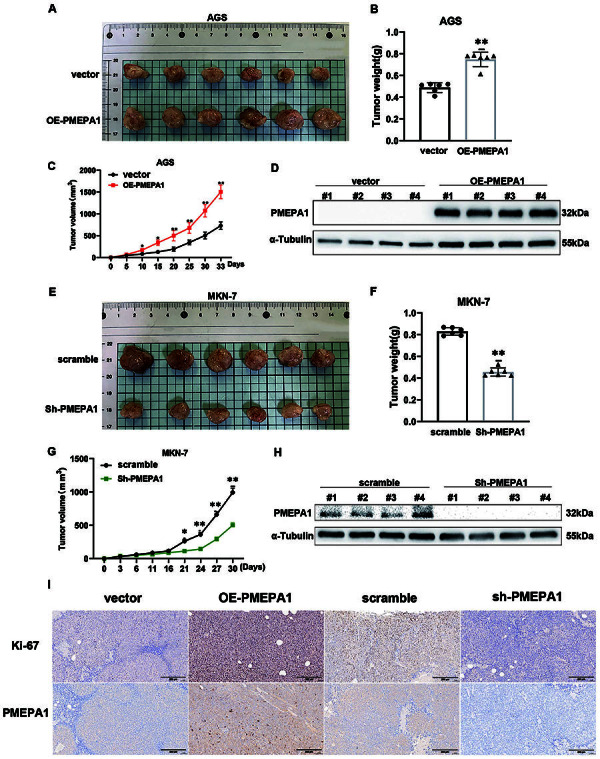
Validation of the oncogenic function of PMEPA1 *in vivo*. PMEPA1-overexpressing AGS cells, PMEPA1-silenced MKN-7 cells, and their respective control cells were subcutaneously injected into NOD-SCID mice and then the mice were sacrificed, and tumors were measured and are shown at post-30-day (**A** and **E**). Tumor weights were recorded post-excision (**B** and **F**). Tumor volumes were measured every 5 days, generating growth curves (**C** and **G**). Tumor tissues were analyzed for PMEPA1 expression via western blot (**D** and **H**), and a portion was immunohistochemically examined for PMEPA1 and Ki67 expression (**I**, scale bar 200 μm). Data are reported as means±SD. *P<0.05, **P<0.01 compared to vector or scramble group; Student's *t*-test.

*PMEPA1*-overexpressing AGS cells, *PMEPA1*-silenced MKN-7 cells, and their respective control cells were subcutaneously injected into NOD-SCID mice to evaluate tumor growth. The *PMEPA1* overexpression group demonstrated faster tumor growth, as evidenced by the increased tumor size and weight (Figure 3A-C), while the *PMEPA1* knockdown group showed reduced tumor growth and weight (Figure 3E-G). After tumor excision, Western blot analysis was performed, confirming PMEPA1 expression (Figure 3D and H). *PMEPA1* overexpression promoted PMEPA1 expression, while *PMEPA1* knockdown inhibited PMEPA1 expression. Immunohistochemical staining of tissue sections indicated that higher PMEPA1 expression was correlated with higher Ki67 expression (Figure 3I).

### Transcriptome sequencing revealed that PMEPA1 binds to the key cell cycle checkpoint protein 14-3-3**σ**

**Figure 4:**
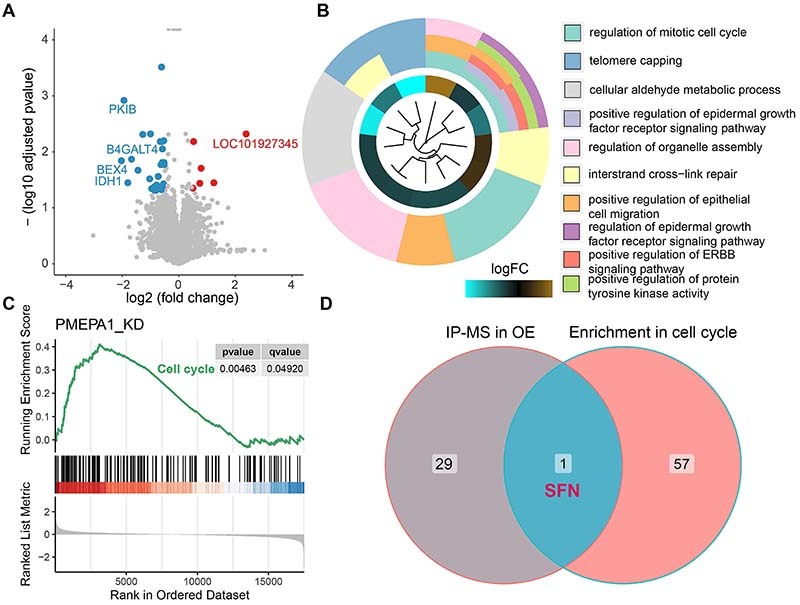
Transcriptome sequencing analysis and pathway enrichment analysis. Volcano plot illustrating the results of differential gene analysis between the PMEPA1 knockdown group and the control group of MKN-7 cells in transcriptome sequencing (**A**). GO enrichment analysis of differentially expressed genes reveals prominent enrichment in the biological process of cell cycle (**B**). Gene Set Enrichment Analysis (GSEA) indicated predominant upregulation of gene expression in the cell cycle pathway, with prominent representation among the top-ranked differentially expressed genes (**C**). The intersection of proteins detected exclusively in the overexpression group through mass spectrometry analysis and the set of differentially expressed genes yielded the identification of SFN (14-3-3σ) (**D**).

The *PMEPA1* knockdown and control groups of MKN-7 cells were subjected to transcriptome sequencing, which revealed 174 differentially expressed genes (139 downregulated, 35 upregulated; Supplementary Table S2, log_2_FC >1, P<0.05) (Figure 4A). KEGG pathway and GO enrichment analyses were subsequently performed on these genes (Supplementary Figure S3A and B), and these analyses consistently revealed enrichment of the cell cycle pathway. The results of GO biological process (BP) analysis are shown in Figure 4B. Furthermore, gene set enrichment analysis (GSEA) revealed that genes within the “cell cycle” pathway were enriched among the differentially expressed genes (Figure 4C).

To identify proteins that interact with PMEPA1, IP was performed on extracts from AGS cells of both the control and *PMEPA1*-overexpression groups using an anti-HA antibody. The immunoprecipitates were then analyzed by mass spectrometry. The analysis identified 227 proteins in the control group and 174 in the *PMEPA1*-overexpression group (Supplementary Tables S3 and S4), with 30 unique to the *PMEPA1*-overexpression group (Supplementary Table S5), indicating their potential interaction with PMEPA1. By taking the intersection between these 30 proteins and the genes in the cell cycle pathway in the KEGG database, 14-3-3σ (SFN) was identified as both a cell cycle gene and a protein interacting with exogenous PMEPA1 (Figure 4D).

### The PMEPA1/14-3-3**σ**/Cdc25c signaling axis altered the proliferation of gastric cancer cells by affecting the cell cycle

**Figure 5:**
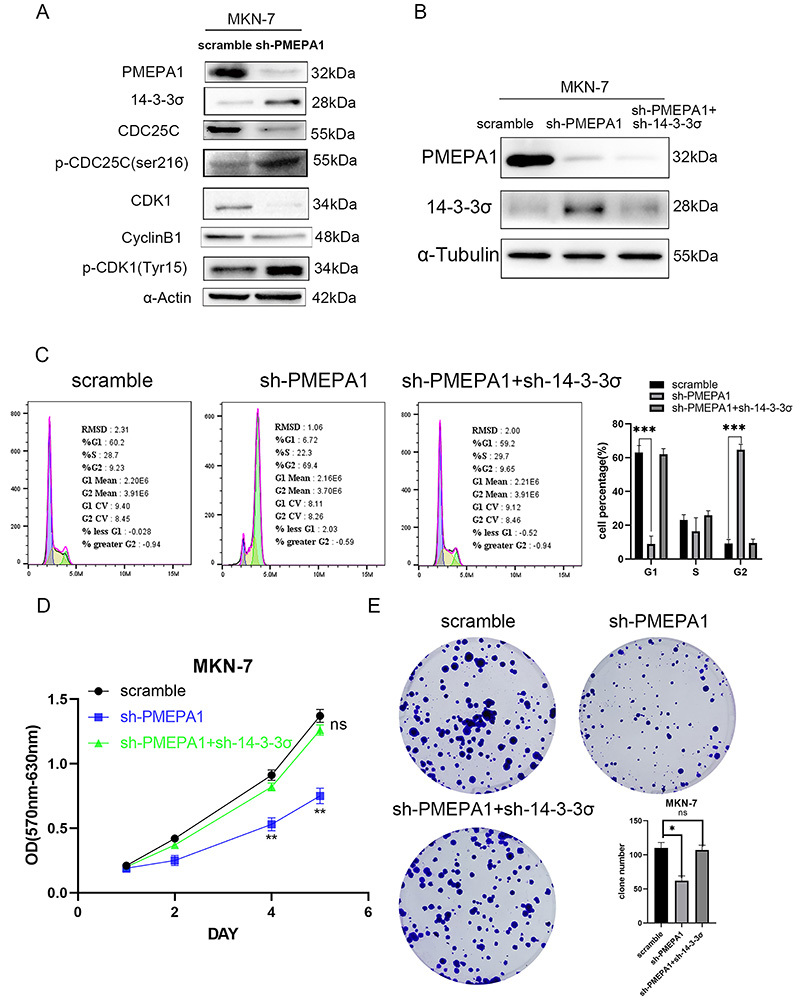
PMEPA1 influenced cell proliferation and cell cycle through the 14-3-3σ/cdc25c signaling axis. After knocking down PMEPA1 in MKN-7 cells, changes in the expression and phosphorylation levels of 14-3-3σ, downstream Cdc25c, and the cdk1/cyclin B complex were observed by western blot (**A**). The shRNA vector of PMEPA1 and 14-3-3σ were transfected into MKN-7 cells, and the expression levels of PMEPA1 and 14-3-3σ proteins were detected by western blot (**B**). The cell cycle distribution (**C**), cell proliferation ability (**D**), and colony formation capacity (**E**) were assessed after the double knockdown of PMEPA1 and 14-3-3σ using flow cytometry, MTT, and clone formation assays, respectively. Data are reported as means±SD. *P<0.05, **P<0.01, ***P<0.001, ns: non-significant compared to scramble group; Student's *t*-test.

The expression of several important checkpoint proteins involved in the G2/M transition was examined to investigate their roles. *PMEPA1* knockdown increased the phosphorylation of Cdc25c at Ser216, reduced the activation of Cdc25c, and maintained a high level of cdk1 phosphorylated at Tyr15, indicating that cdk1 was not dephosphorylated by Cdc25c (Figure 5A). Increased expression levels of 14-3-3σ were also noted after *PMEPA1* knockdown. 14-3-3σ binds to Cdc25c, maintaining its phosphorylation at serine 216 and thereby inhibiting Cdc25c activity, which prevents cell cycle progression and leads to G2/M arrest (20). Furthermore, 14-3-3σ can suppress the activity of the cdk1-cyclinB1 complex, contributing to cell cycle arrest (21). To further elucidate the role of 14-3-3σ in G2/M arrest following *PMEPA1* knockdown, rescue experiments were conducted by establishing cells with double knockdown of *PMEPA1* and *14-3-3σ*. The transfection efficiency was determined (Figure 5B). sh-*PMEPA1*-induced G2/M arrest was reversed by *14-3-3σ* knockdown. Additionally, there was no significant difference in the proportion of G2-phase cells between the double knockdown group and the control group, with percentages of 9.65 and 9.23%, respectively (Figure 5C), suggesting that the G2/M arrest caused by the upregulation of 14-3-3σ due to *PMEPA1* knockdown is reversible. Moreover, the proliferation capacity of the *PMEPA1*/*14-3-3σ* double knockdown cells was restored to the level of the control cells (Figure 5D and E).

### PMEPA1 bound to 14-3-3**σ** and promoted its degradation by facilitating its ubiquitination

**Figure 6:**
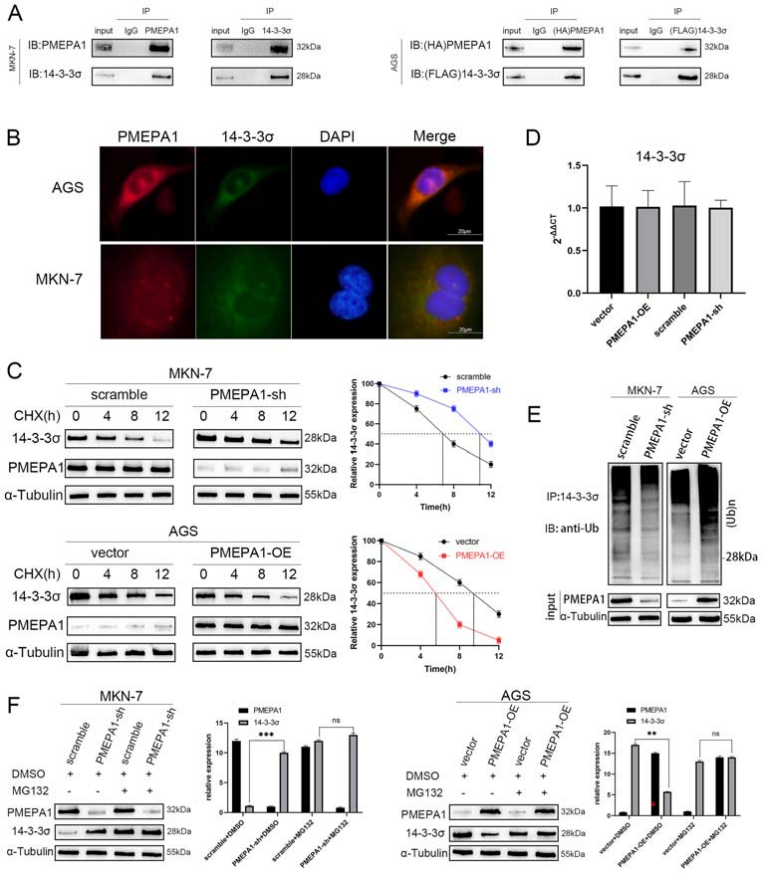
PMEPA1 interacted with 14-3-3σ and affected its stability. In AGS cells (exogenous) and MKN-7 cells (endogenous), the interaction between PMEPA1 and 14-3-3σ was validated using co-immunoprecipitation (co-IP) (**A**). The interaction between PMEPA1 and 14-3-3σ in AGS and MKN-7 cells was further confirmed through immunofluorescence assays (**B**, scale bar 20 μm). The stability of 14-3-3σ expression following overexpression or knockdown of PMEPA1 in AGS or MKN-7 cells was assessed by western blot after treating the cells with cycloheximide (CHX, 20 μg/mL) for 0, 4, 8, and 12 h (**C**). The changes in mRNA levels of 14-3-3σ after overexpression or knockdown of PMEPA1 in AGS or MKN-7 cells were detected by quantitative PCR (qPCR) (**D**). The impact of overexpression or knockdown of PMEPA1 on the ubiquitination levels of 14-3-3σ was examined through co-IP (**E**). After treating the PMEPA1 overexpression AGS cells or PMEPA1 knockdown MKN-7 cells with MG132 (25 μM) for 24 h, the reversal of 14-3-3σ degradation was evaluated by western blot (**F**). Data are reported as means±SD. **P<0.01, ***P<0.001, ns: non-significant; Student's *t*-test.

Given the critical role of 14-3-3σ in cell cycle checkpoints and its intimate involvement in cell cycle alterations following the knockdown of *PMEPA1*, we continued to explore how PMEPA1 interacts with 14-3-3σ and affects its expression level. The results of the coimmunoprecipitation (Co-IP) assay confirmed the physical interaction between endogenous 14-3-3σ and PMEPA1 in MKN-7 cells (Figure 6A). Additionally, exogenous 14-3-3σ was shown to interact with exogenous PMEPA1 in AGS cells co-transfected with the *14-3-3σ* and *PMEPA1* expression plasmids (Figure 6A). Immunofluorescence staining demonstrated the colocalization of 14-3-3σ and PMEPA1 in both AGS cells (with exogenous expression) and MKN-7 cells (with endogenous expression) (Figure 6B). Overall, these findings provided evidence of the interaction between 14-3-3σ and PMEPA1.

Next, the effect of PMEPA1 on the stability of the 14-3-3σ protein after inhibition of protein synthesis with cycloheximide was analyzed. In MKN-7 cells with *PMEPA1* knockdown, 14-3-3σ was degraded rapidly, whereas in AGS cells with *PMEPA1* overexpression, the half-life of 14-3-3σ was extended (Figure 6C). However, the mRNA level of 14-3-3σ remained constant (Figure 6D), indicating that PMEPA1 affects the protein stability of 14-3-3σ. Additionally, the reduction in the 14-3-3σ protein level in *PMEPA1*-overexpressing cells was specifically reversed by treatment with the proteasome inhibitor MG132 (Figure 6F), suggesting that the mechanism by which PMEPA1 promoted the instability of 14-3-3σ was dependent on the proteasome.

Subsequently, the mechanism by which PMEPA1 reduces the stability of 14-3-3σ was investigated. Since ubiquitination is a key event driving proteasomal degradation (22), the ability of PMEPA1 modulation to regulate the ubiquitination of 14-3-3σ was assessed. *PMEPA1* knockdown decreased but *PMEPA1* overexpression increased 14-3-3σ ubiquitination (Figure 6E). This indicates that PMEPA1 bound to 14-3-3σ, promoting its ubiquitination and subsequent degradation through the ubiquitin-proteasome pathway in gastric cancer cells.

### PMEPA1 recruited TTC3, allowing the ubiquitination of 14-3-3**σ** and promoting its ubiquitin-mediated degradation.

**Figure 7:**
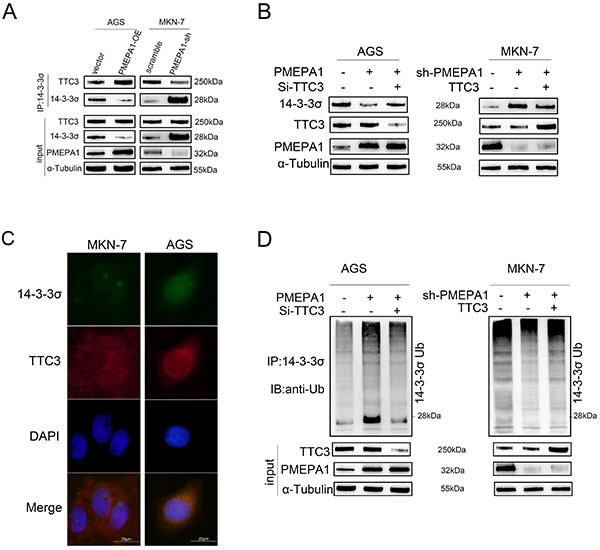
PMEPA1 recruited TTC3 to facilitate the ubiquitination of 14-3-3σ. Co-IP assays demonstrated the impact of PMEPA1 expression changes on the binding of TTC3 to 14-3-3σ in AGS and MKN-7 cells (**A**). Immunoblotting revealed that TTC3 siRNA reversed the degradation level of 14-3-3σ following the overexpression of PMEPA1. Moreover, even with the overexpression of TTC3, the knockdown of PMEPA1 also reversed the degradation level of 14-3-3σ (**B**). Immunofluorescence staining indicated that 14-3-3σ can bind with TTC3 (**C**, scale bar 20 μm). Co-IP assays showed that siTTC3 reversed the ubiquitination of 14-3-3σ following the overexpression of PMEPA1, and similarly, even with the overexpression of TTC3, the knockdown of PMEPA1 also reversed the ubiquitination of 14-3-3σ (**D**).

Through immunoprecipitation-mass spectrometry (IP-MS) analysis, we identified TTC3 as a protein that interacted with PMEPA1 (Supplementary Table S4). TTC3, an E3 ubiquitin ligase, is known for its role in the ubiquitin-mediated degradation of phosphorylated AKT, which terminates AKT pathway activation (23), and for its role in ubiquitinating SMURF2 to activate the TGF-β pathway, in turn promoting epithelial-mesenchymal transition (EMT) and myofibroblast differentiation (24). The known important role of PMEPA1 in the TGF-β pathway implies that TTC3 may interact with PMEPA1. Hence, whether PMEPA1 recruited TTC3 to bind to 14-3-3σ in gastric cancer cells was investigated. Overexpression of *PMEPA1* in AGS cells promoted the interaction between TTC3 and 14-3-3σ and decreased 14-3-3σ expression, while *PMEPA1* knockdown in MKN-7 cells had the opposite effects (Figure 7A). The colocalization of 14-3-3σ and TTC3 was confirmed in both AGS cells (with exogenous expression) and MKN-7 cells (with endogenous expression) (Figure 7C). Knocking down *TTC3* in *PMEPA1*-overexpressing AGS cells restored 14-3-3σ degradation (Figure 7B) and decreased its ubiquitination (Figure 7D). However, overexpressing *TTC3* in *PMEPA1* knockdown MKN-7 cells did not result in significant degradation of 14-3-3σ (Figure 7B) and did not significantly increase its ubiquitination (Figure 7D), indicating the necessity of PMEPA1 for TTC3-mediated ubiquitination of 14-3-3σ. In summary, these results suggested that PMEPA1 recruited TTC3, allowing it to bind 14-3-3σ and promote its ubiquitin-mediated degradation in gastric cancer cells.

## Discussion

Gastric cancer constitutes a substantial proportion of the global disease burden, particularly in East Asia (25). Despite the considerable advancements in systemic therapy for gastric cancer, the overall survival rate of patients with this disease remains low. Chemotherapy is a crucial component of systemic therapeutic strategies for gastric cancer, and the mechanism of action of chemotherapeutic drugs is often closely associated with the cell cycle. Our findings underscored the critical role of *PMEPA1* in the cell cycle, thus influencing gastric cancer cell proliferation.

Regarding the specific mechanisms by which the cell cycle is affected, ectopic PMEPA1 overexpression has been reported to promote the proliferation of androgen receptor-negative prostate cancer cells by inhibiting p21 (26). Moreover, a study on triple-negative breast cancer reported that knocking down PMEPA1 increased p27 expression (27). Both p21 and p27 belong to the CDK-interacting protein/kinase inhibitory protein (cip/kip) family, whose members can broadly inactivate cyclin-CDK complexes. p21 is activated by p53 (28) in response to DNA damage. p27 is activated by transforming growth factor β (TGF-β) (29), a growth inhibitor. These observations suggest that PMEPA1 affects the cell cycle of tumor cells.

We conducted transcriptome sequencing on samples with *PMEPA1* knockdown, and subsequent pathway enrichment analysis of the differentially expressed genes revealed a significant effect on the cell cycle pathway. IP-MS analysis of *PMEPA1*-overexpressing samples revealed that PMEPA1 can bind to the key cell cycle checkpoint protein 14-3-3σ. Both 14-3-3σ and Cdc25c are key proteins in cell cycle checkpoints. Cdc25c activates the CDK1-cyclinB complex by dephosphorylating Tyr15 of CDK1 (30), thereby promoting cell cycle progression. 14-3-3σ can phosphorylate Cdc25 at Ser216 (20), leading to its inactivation and resulting in G2/M arrest. Known for its role in the G2/M transition (21), 14-3-3σ can prevent the nuclear entry of CDK1 (31), inhibit cell cycle progression, and cause dissociation of the CDK1-cyclinB complex (32). This complex can potentially be regulated via two pathways. The first pathway is the P53/14-3-3σ pathway (21), in which P53 promotes the expression of 14-3-3σ, leading to the cytoplasmic sequestration or the dissociation of the cdk1-cyclinB complex, thereby inhibiting its activity. The second pathway is the chk1/cdc25c pathway, in which yeast checkpoint kinases, specifically chk1, are involved in DNA damage checkpoint responses. Studies have shown that chk1 can phosphorylate cdc25c at Ser216 *in vitro*, leading to the binding of phosphorylated cdc25c to 14-3-3σ, in turn resulting in the loss of cdc25c activity (20). However, cdc25c can be activated by dissociation from 14-3-3σ and dephosphorylation at Ser216, and activated cdc25c then dephosphorylates Tyr15 of cdk1 (33), activating cdk1. Furthermore, the cdk1/cyclinB1 complex can phosphorylate cdc25c at several sites, including Thr48, Thr67, Thr138, Ser205, and Ser285, facilitating positive feedback activation of cdc25c and promoting cell cycle progression (30). In our study, sh-*PMEPA1*-induced G2/M arrest in gastric cancer cells was reversed by 14-3-3σ knockdown. In summary, transcriptome and IP-MS analyses revealed that PMEPA1 influenced cell cycle progression by interacting with the critical checkpoint protein 14-3-3σ. This interaction impacted key regulatory pathways, notably reversing G2/M arrest in gastric cancer cells through 14-3-3σ knockdown, identifying potential targets for cancer therapy.

By investigating the specific mechanism by which PMEPA1 acts on 14-3-3σ, we identified TTC3, an E3 ubiquitin ligase, among the proteins that interact with PMEPA1. Tetratricopeptide Repeat Domain 3 (TTC3) is an ubiquitin E3 ligase that can induce the ubiquitination and proteasomal degradation of SMAD ubiquitination regulatory factor 2 (SMURF2) (24). This process facilitates TGF-β1-induced EMT and myofibroblast differentiation. TTC3 has been reported to catalyze the formation of Lys48-linked polyubiquitin chains, mediating the ubiquitination and degradation of phosphorylated Akt in the nucleus (23,24). Additionally, TTC3 has been reported to inhibit cell proliferation in human cell lines (34). We investigated the relationships between the ubiquitination level of 14-3-3σ and the expression levels of PMEPA1 and TTC3 and discovered that PMEPA1 can recruit TTC3, allowing the ubiquitination and degradation of 14-3-3σ and thereby facilitating cell cycle progression. When *PMEPA1* is knocked down, 14-3-3σ ubiquitination decreases, leading to an increase in the 14-3-3σ protein level and inducing G2/M arrest in gastric cancer cells.

### Conclusion

In this study, we discovered that PMEPA1 promoted tumor progression, particularly by increasing tumor cell proliferation. Knocking down *PMEPA1* induced cell cycle arrest. The underlying mechanism involves the recruitment of the E3 ubiquitin ligase TTC3 by PMEPA1, which allows the ubiquitination and degradation of 14-3-3σ. Following *PMEPA1* knockdown, the stability of 14-3-3σ increased, and its degradation rate decreased, inhibiting cell cycle progression and leading to G2/M arrest.

## Supplementary Material

Click here to view [pdf].
