# Supplementary material for: PMEPA1 promotes gastric cancer cell proliferation by regulating the ubiquitin-mediated degradation of 14-3-3σ and promoting cell cycle progression
Source: Braz J Med Biol Res. 2024 Nov 25;57:e13985. doi: 10.1590/1414-431X2024e13985 (PMC11653473; doi:10.1590/1414-431X2024e13985)
Supplement: Supplementary file 1 [file 1414-431X-bjmbr-57-e13985-suppl.pdf]

Figure S1. Transcriptome sequencing of collected gastric cancer and adjacent non-tumor specimens. **A**, The heatmap analysis of transcriptome sequencing was performed on paired tumor and adjacent normal tissue samples from gastric cancer patients. The top 100 differentially expressed genes were selected for visualization. **B**, Differential gene volcano plot analysis revealed that *PMEPA1* is located in the "upregulated" group with the log_2FC value=3.398 and the P-value=0.000595.

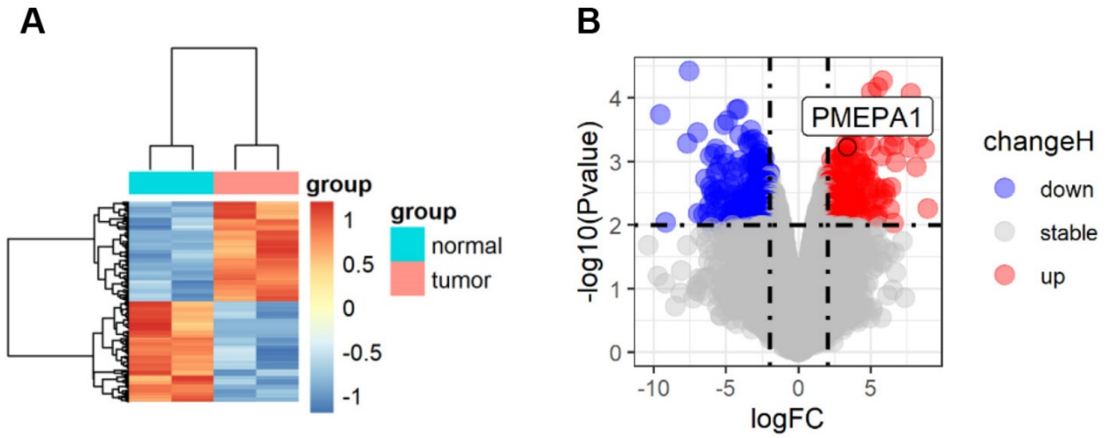

Figure S2. The expression level of PMEPA1 in gastric cancer cell lines. Western blot assay was applied to detect the protein level of PMEPA1 in human gastric epithelial cells GSE-1 and human gastric cancer cell lines (AGS, BGC-823, HGC-27, MKN-7, MKN-45).

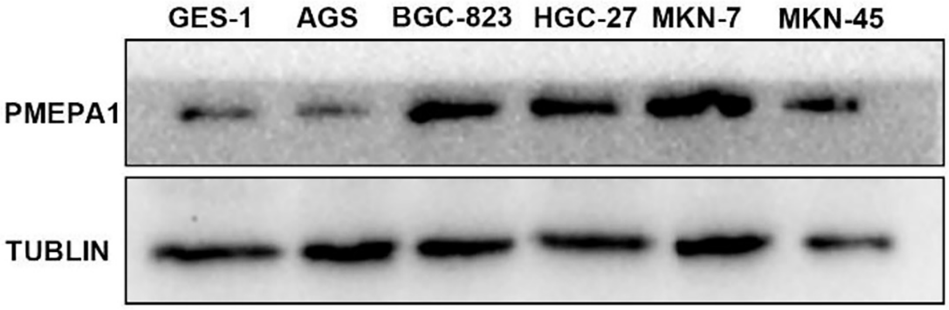

Figure S3. KEGG pathway and GO enrichment of 174 differentially expressed genes in PMEPA1 knockdown and control groups of MKN-7 cell. **A**, Following transcriptome sequencing of the *PMEPA1* knocked-down cell line and the control group, KEGG pathway analysis was conducted. The enrichment analysis results indicated that the cell cycle pathway ranked third in terms of enrichment. **B**, In the GO analysis of biological process (BP) category, the cell cycle pathway was found to be enriched and ranked second in terms of enrichment among the gene groups from the knocked-down cell line and the control group.

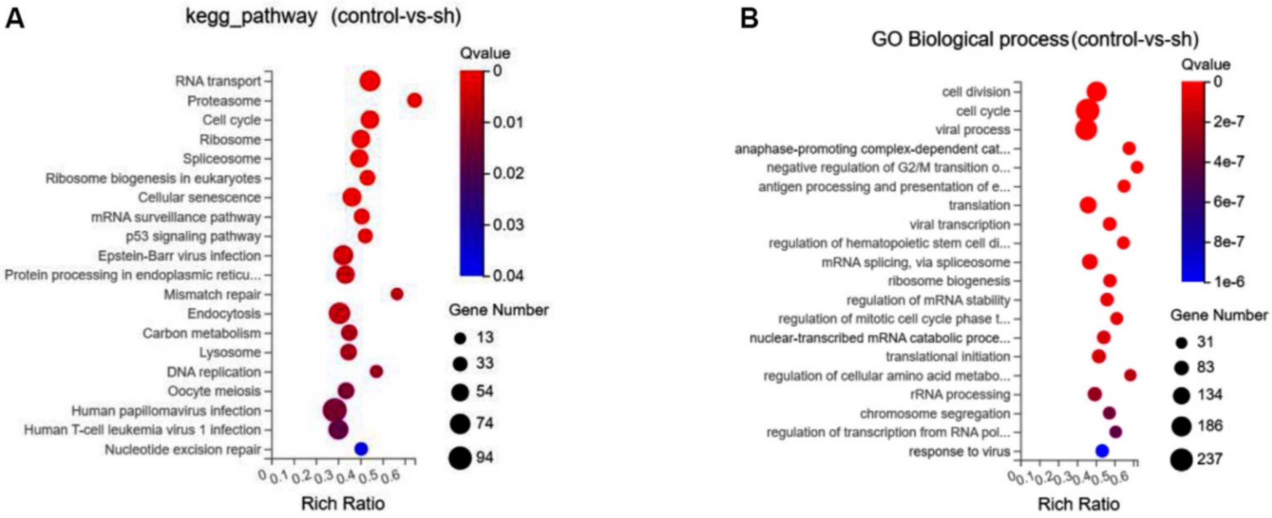

Table S1. Primers used in plasmid construction.

sh-PMEPA1	
Sense	5'-CCGGGAGCAAAGAGAAGGATAAACACTCGAGTGTTTATCCTTCTCTTTGCTCTTTTTG-3'
Anti-sense	5'-AATTCAAAAAGAGCAAAGAGAAGGATAAACACTCGAGTGTTTATCCTTCTCTTGCTC-3'
sh-14-3-3σ	
Sense	5'-CCGGACCTGAAGATGAAGGGTGACTCTCGAGAGTCACCCTTCATCTTCAGGTTTTTTG-3'
Anti-sense	5'-AATTCAAAAAACCTGAAGATGAAGGGTGACTCTCGAG AGTCACCCTTCATCTTCAGGT-3'
Scrambled shRNA	
Sense	5'-GATCCAACAAGATGAAGAGCACCAACTCGAGTTGGTGCTCTTCATCTTGTTGTTTTTG-3'
Anti-sense	5'-AATTCAAAAACAACAAGATGAAGAGCACCAACTCGAGTTGGTGCTCTTCATCTTGTTG-3'
OE-PMEPA1 (HA)	
Sense	5'GGGGTACCGCCACCATGTACCCATACGATGTTCCAGATTACGCTGGTGGAGGCGGTAGCCACCGCTTGATGGGG-3'
Anti-sense	5'-CCGCTCGAGCTAGAGAGGGTGTCCTT-3'
Exogenous-14-3-3σ (FLAG)	
Sense	5'-CGGAATTCGCCACCATGGAGAGAGCCAGTCTGAT-3'
Anti-sense	5'-CCGCTCGAGTCACTTGTCATCGTCGTCCTTGTAATCGCTCTGGGGCTCCT-3'

Table S2. List of the 174 differentially expressed genes in PMEPA1 knockdown and control groups of MKN-7 cells.

st_gene_id	gene_id	Diffexp_gene	log2fc_control-vs-sh	deseq2_qvalue	deseq2_pvalue
G9606_5583	8061	FOSL1	1.688375245	3.14064E-83	3.27282E-86
G9606_13999	135932	TMEM139	1.677358649	2.52092E-09	6.50917E-11
G9606_24940	2152	F3	2.128897687	1.1837E-105	8.2238E-109
G9606_10909	23022	PALLD	1.603245187	4.1446E-105	3.1193E-108
G9606_26247	25805	BAMBI	1.598618444	1.44576E-13	2.05903E-15
G9606_19033	23767	FLRT3	1.522134511	0.003810956	0.000465752
G9606_3098	1368	CPM	1.62714045	2.53694E-25	1.68904E-27
G9606_30449	125965	COX6B2	1.606845412	2.53775E-05	1.5118E-06
G9606_4253	91662	NLRP12	1.908129445	0.005951946	0.000790839
G9606_3583	4008	LMO7	2.666372985	6.6676E-118	3.8601E-121
G9606_15127	3910	LAMA4	1.512239831	1.94856E-08	5.80051E-10
G9606_1752	285	ANGPT2	4.745993032	1.28117E-59	2.29932E-62
G9606_6511	54221	SNTG2	3.034506981	1.28262E-10	2.66579E-12
G9606_23085	1961	EGR4	2.212363179	0.003680887	0.000447298
G9606_2833	132671	SPATA18	3.554874909	1.61713E-05	9.07197E-07
G9606_7396	29091	STXBP6	2.890003744	9.82401E-20	8.70186E-22
G9606_12862	101059949	LOC101059949	3.35707906	0.009911193	0.001436787
G9606_31325	6781	STC1	6.012497684	0.006241791	0.000838358
G9606_4540	1960	EGR3	1.813396571	5.0534E-07	1.96016E-08
G9606_6704	3038	HAS3	1.708556836	1.437E-101	1.1647E-104
G9606_6274	129293	TRABD2A	1.856149276	1.82185E-09	4.60921E-11
G9606_17206	860	RUNX2	2.04878913	1.1978E-11	2.19131E-13
G9606_5220	56994	CHPT1	1.800253033	3.52214E-20	2.99235E-22
G9606_13338	101927345	LOC101927345	3.013146744	1.30475E-07	4.56244E-09
G9606_11801	375616	KCP	1.60723259	5.07369E-05	3.26634E-06
G9606_6408	10761	PLAC1	1.829047301	0.002433575	0.000277269
G9606_22689	4488	MSX2	1.537452334	1.91366E-20	1.59536E-22
G9606_24273	1880	GPR183	2.32661196	0.001460563	0.000153641
G9606_8923	650	BMP2	3.136860463	0.001333003	0.000137059
G9606_9300	639	PRDM1	2.113311805	7.27582E-18	7.2872E-20
G9606_18170	51200	CPA4	1.537624029	2.39796E-39	8.46846E-42
G9606_23083	112267876	LOC112267876	2.321280735	3.55739E-05	2.20574E-06
G9606_14931	1462	VCAN	1.951821262	3.3395E-124	1.5467E-127
G9606_35932	6591	SNAI2	1.545346274	0.000145233	1.08464E-05
G9606_16971	1800	DPEP1	3.418728104	6.42311E-66	9.29646E-69
G9606_20444	2159	F10	-3.043189834	0.000129792	9.58054E-06
G9606_10534	19	ABCA1	-1.60335535	0.00242852	0.000276272
G9606_25650	282763	OR51B5	-1.640324239	8.47019E-31	4.11912E-33
G9606_4729	10231	RCAN2	-1.737085668	0.004526543	0.00057155
G9606_29651	64135	IFIH1	-2.338553417	9.57064E-55	2.0501E-57
G9606_985	10561	IFI44	-5.812689107	1.16763E-19	1.04102E-21
G9606_17326	116071	BATF2	-3.54736277	3.06652E-21	2.44995E-23
G9606_12539	4940	OAS3	-2.479773058	1.0873E-187	2.5179E-191
G9606_2530	83546	RTBDN	-1.641780346	1.93263E-10	4.12863E-12
G9606_5998	266977	ADGRF1	-1.594798526	2.42494E-39	8.70413E-42
G9606_19164	710	SERPING1	-5.924379345	2.7143E-06	1.26408E-07
G9606_24622	55337	SHFL	-2.697233476	8.87902E-65	1.38791E-67
G9606_25011	54809	SAMD9	-2.984892405	1.4002E-149	4.0533E-153
G9606_25062	2172	FABP6	-1.557807973	0.000926292	9.02773E-05
G9606_24332	151636	DTX3L	-2.028598319	1.94664E-36	7.77617E-39
G9606_7611	2122	MECOM	-1.505341881	0.000168724	1.28201E-05
G9606_28539	12	SERPINA3	-1.96921858	0.004090125	0.000505079
G9606_13998	27128	CYTH4	-1.578632026	1.38825E-05	7.61805E-07
G9606_12746	445582	POTEE	-6.144558838	0.005569301	0.000729332
G9606_14000	5610	EIF2AK2	-1.59678594	7.15176E-63	1.15932E-65
G9606_401	5104	SERPINA5	-1.919865326	0.000297158	2.46184E-05
G9606_10981	3429	IFI27	-6.554771387	8.52441E-39	3.15847E-41
G9606_22285	6772	STAT1	-2.579439842	7.1621E-278	4.1464E-282
G9606_7109	84632	AFAP1L2	-3.621454824	2.43464E-05	1.43911E-06
G9606_7658	54875	CNTLN	-2.103158559	1.41798E-13	2.01126E-15
G9606_5736	10964	IFI44L	-6.788670906	0.000479455	4.258E-05
G9606_23166	5631	PRPS1	-1.797384829	2.25192E-53	5.21301E-56
G9606_1670	1742	DLG4	-1.503619445	1.8251E-13	2.63098E-15
G9606_19917	9915	ARNT2	-1.80988625	5.88124E-08	1.92716E-09
G9606_9861	4938	OAS1	-5.210890909	6.19427E-52	1.54202E-54
G9606_34264	6192	RPS4Y1	-8.680135034	1.96489E-07	7.15517E-09
G9606_2108	9636	ISG15	-3.221097961	7.4081E-254	8.5777E-258
G9606_24699	100507003	GFY	-3.582584726	8.80455E-21	7.18718E-23
G9606_15553	400793	C1orf226	-1.501594071	3.2301E-08	1.01355E-09
G9606_14035	10699	CORIN	-1.734576634	0.000675633	6.2936E-05
G9606_1903	8638	OASL	-3.078386697	2.00082E-38	7.5293E-41
G9606_1105	22891	ZNF365	-1.506154751	0.00085339	8.22116E-05
G9606_19783	53840	TRIM34	-4.343685256	8.053E-15	9.83722E-17
G9606_8457	3965	LGALS9	-2.140720799	0.000117517	8.50441E-06
G9606_12236	85441	HELZ2	-2.020511727	6.83222E-82	7.51532E-85
G9606_16238	2678	GGT1	-1.746595173	5.31204E-15	6.3352E-17
G9606_14762	6920	TCEA3	-1.901339642	8.60425E-07	3.53176E-08
G9606_6788	6304	SATB1	-1.888714244	5.37887E-10	1.23627E-11

G9606_9268	83666	PARP9	-2.893328988	8.8945E-127	3.6046E-130
G9606_33670	388115	CCDC9B	-1.708541826	4.72868E-15	5.61211E-17
G9606_11889	5570	PKIB	-1.941154526	9.13612E-28	5.28925E-30
G9606_5137	22914	KLRK1	-2.106727713	1.47061E-05	8.1478E-07
G9606_9448	79132	DHX58	-2.643329465	0.008894221	0.001270434
G9606_32220	7098	TLR3	-2.02203318	2.53241E-08	7.68241E-10
G9606_5315	140738	TMEM37	-2.00999419	0.004618626	0.000587456
G9606_10235	114336	CGB2	-1.605575779	1.40426E-05	7.75581E-07
G9606_22493	6947	TCN1	-2.385723524	0.002247662	0.000251663
G9606_19510	2811	GP1BA	-2.13130174	0.00030154	2.50512E-05
G9606_24182	84875	PARP10	-3.687961183	5.09189E-18	5.01142E-20
G9606_28657	3433	IFIT2	-3.80478767	7.7658E-34	3.46186E-36
G9606_21061	3745	KCNB1	-2.097613221	0.001980457	0.000217803
G9606_17881	9619	ABCG1	-1.555440392	0.000190195	1.48166E-05
G9606_10589	8702	B4GALT4	-1.920015873	2.24536E-25	1.48191E-27
G9606_12604	340277	FAM221A	-2.676151252	0.002232559	0.000249843
G9606_30798	10410	IFITM3	-1.592683194	6.68898E-75	7.74501E-78
G9606_20994	684	BST2	-3.662730814	3.36942E-39	1.22893E-41
G9606_6051	25837	RAB26	-1.915980879	1.05887E-16	1.1157E-18
G9606_19960	129607	CMPK2	-2.838136436	1.18753E-08	3.39628E-10
G9606_5729	407977	TNFSF12-TNFSF13	-6.287254883	0.002326065	0.000263135
G9606_1770	10202	DHRS2	-2.77563784	1.33016E-93	1.15512E-96
G9606_26416	7991	TUSC3	-6.329623445	0.002163673	0.000240631
G9606_13848	837	CASP4	-2.522129199	1.66852E-55	3.38089E-58
G9606_30118	6275	S100A4	-1.983076844	7.6318E-46	2.16499E-48
G9606_29597	11067	DEPP1	-2.643206993	8.78318E-07	3.61029E-08
G9606_3987	10371	SEMA3A	-1.578927222	1.89369E-17	1.91858E-19
G9606_9498	23780	APOL2	-1.528259108	5.21035E-07	2.0331E-08
G9606_1939	168544	ZNF467	-1.597663727	2.55094E-07	9.46653E-09
G9606_32476	6398	SECTM1	-1.566531661	1.40621E-09	3.49252E-11
G9606_14083	94240	EPSTI1	-3.681573409	1.13485E-14	1.41914E-16
G9606_16708	2537	IFI6	-6.353312304	3.8828E-116	2.4727E-119
G9606_35294	440836	ODF3B	-1.51349185	0.00058212	5.31804E-05
G9606_17995	51702	PADI3	-2.86287808	0.005297226	0.000688182
G9606_1460	79633	FAT4	-1.578501249	0.001414	0.000147351
G9606_7945	79339	OR51B4	-1.615354278	9.40339E-10	2.27558E-11
G9606_30845	79345	OR51B2	-1.634801808	5.72009E-05	3.74539E-06
G9606_5528	55008	HERC6	-1.899327562	1.1243E-33	5.07702E-36
G9606_1548	5359	PLSCR1	-1.626027216	5.78921E-60	1.00548E-62
G9606_12091	114990	VASN	-1.77293995	0.000181787	1.405E-05
G9606_12740	55601	DDX60	-2.578842878	2.83549E-74	3.44731E-77
G9606_17146	10475	TRIM38	-1.529595361	6.64802E-43	2.03986E-45
G9606_10696	219285	SAMD9L	-3.349114864	0.00236837	0.000268195
G9606_25759	2254	FGF9	-2.202084305	2.29254E-05	1.34762E-06
G9606_3573	6773	STAT2	-1.670418057	4.45606E-41	1.44468E-43
G9606_1634	7786	MAP3K12	-1.679538581	2.95298E-14	3.7782E-16
G9606_20647	3437	IFIT3	-4.215415037	1.57937E-35	6.49196E-38
G9606_27267	56241	SUSD2	-1.792371084	1.0247E-08	2.88314E-10
G9606_24247	9086	EIF1AY	-5.915044873	0.007382462	0.001023193
G9606_17948	6402	SELL	-1.631659971	0.000255691	2.06797E-05
G9606_10600	8497	PPFIA4	-1.659080623	2.2379E-06	1.02094E-07
G9606_16819	1299	COL9A3	-1.685773054	4.75559E-23	3.49412E-25
G9606_6988	56937	PMEPA1	-2.10639654	0.000634613	5.85638E-05
G9606_12703	3665	IRF7	-2.584290677	2.29891E-85	2.26258E-88
G9606_22069	56924	PAK6	-2.050527797	9.66503E-05	6.7761E-06
G9606_30602	56271	BEX4	-3.240446794	2.22353E-05	1.30145E-06
G9606_17933	5745	PTH1R	-6.028958273	0.00592543	0.000785575
G9606_447	27239	GPR162	-1.76888689	0.005533114	0.000723312
G9606_8327	84080	ENKD1	-2.763990276	4.33295E-23	3.13564E-25
G9606_14056	6564	SLC15A1	-2.068205529	4.28181E-05	2.70944E-06
G9606_24235	7447	VSNL1	-1.618564788	0.000168071	1.27564E-05
G9606_2441	8653	DDX3Y	-4.086113698	0.001330965	0.000136695
G9606_6644	93273	LEMD1	-1.819663801	0.001488473	0.00015718
G9606_8844	9985	REC8	-2.5560948	2.30897E-13	3.36861E-15
G9606_15023	3860	KRT13	-1.946096795	0.000650689	6.03111E-05
G9606_14781	4781	NFIB	-2.928076886	3.61258E-20	3.11628E-22
G9606_34393	8519	IFITM1	-5.543738565	5.87756E-52	1.42915E-54
G9606_12871	27071	DAPP1	-2.269338968	0.000129004	9.49324E-06
G9606_30184	5698	PSMB9	-2.080410322	1.18828E-16	1.27269E-18
G9606_9059	4599	MX1	-2.598169359	2.40394E-13	3.52108E-15
G9606_4548	7634	ZNF80	-1.763493372	0.003681726	0.000447613
G9606_27001	60681	FKBP10	-1.789822945	2.99397E-58	5.54664E-61
G9606_2782	51513	ETV7	-2.026179404	0.004459036	0.000561219
G9606_11493	85480	TSLP	-2.445172915	9.24598E-06	4.87109E-07
G9606_15179	388611	CD164L2	-1.503763434	0.000947276	9.29013E-05
G9606_5127	23150	FRMD4B	-1.720532711	0.000129952	9.59991E-06
G9606_23698	55534	MAML3	-1.865256487	6.38405E-13	9.83128E-15
G9606_11717	3434	IFIT1	-6.014124411	7.5873E-189	1.3178E-192
G9606_13537	3431	SP110	-2.264508961	1.3775E-46	3.82793E-49
G9606_16753	4066	LYL1	-2.653210096	0.000521074	4.70605E-05

G9606_33671	10379	IRF9	-2.579964306	5.5008E-122	2.8661E-125
G9606_15601	11277	TREX1	-1.777152373	9.97764E-05	7.01837E-06
G9606_32987	6515	SLC2A3	-3.058854017	5.09807E-23	3.77788E-25
G9606_28734	4143	MAT1A	-2.009566408	0.00479177	0.000611629
G9606_15902	9459	ARHGEF6	-1.574530673	5.23157E-06	2.59261E-07
G9606_11076	3417	IDH1	-1.517803723	2.17126E-65	3.26827E-68
G9606_3392	5502	PPP1R1A	-4.026972754	2.3827E-05	1.40565E-06
G9606_2759	1244	ABCC2	-1.68464682	2.1128E-40	7.21675E-43
G9606_12118	2736	GLI2	-3.059389422	1.48533E-11	2.74312E-13
G9606_2921	23089	PEG10	-3.823049769	1.64842E-26	9.92505E-29
G9606_8314	9881	TRANK1	-3.026577362	0.000167993	1.27408E-05
G9606_31781	23586	DDX58	-3.218453191	1.4742E-134	5.1208E-138
G9606_13440	28514	DLL1	-1.594376946	0.000114137	8.22017E-06
G9606_27653	51191	HERC5	-1.909777639	3.56579E-30	1.77536E-32
G9606_14784	9388	LIPG	-1.547879922	5.29935E-05	3.42794E-06
G9606_33859	4856	CCN3	-1.60498891	0.006270768	0.000843359

Table S3. List of the 227 proteins in the control group identified by mass spectrometry.

Accession	Protein names	Gene names	MW [kDa]	Protein score	Sequence coverage (%)	Unique Peptides	Peptides	Abundances
P02545	Prelamin-A/C	LMNA	74.1	1465	42	24	26	1.76e+08
P11021	Endoplasmic reticulum chaperone BiP	HSPA5	72.3	925	35	18	19	1.43e+08
P60709	Actin, cytoplasmic 1	ACTB	41.7	809	38	12	12	3.39e+08
P02768	Albumin	ALB	69.3	770	25	17	17	1.59e+09
P11142	Heat shock cognate 71 kDa protein	HSPA8	70.9	734	23	10	13	8.39e+07
P38646	Stress-70 protein, mitochondrial	HSPA9	73.6	650	25	16	16	6.63e+07
Q86YZ3	Hornerin	HRNR	282.2	462	11	8	8	2.74e+07
P07900	Heat shock protein HSP 90-alpha	HSP90AA1	84.6	462	19	6	13	8.72e+08
P10809	60 kDa heat shock protein, mitochondrial	HSPD1	61	431	14	7	7	3.36e+07
P62701	40S ribosomal protein S4, X isoform	RPS4X	29.6	409	42	15	15	1.22e+08
P07437	Tubulin beta chain	TUBB	49.6	409	19	3	7	2.76e+07
P08238	Heat shock protein HSP 90-beta	HSP90AB1	83.2	392	14	2	10	1.25e+07
P68371	Tubulin beta-4B chain	TUBB4B	49.8	366	15	2	6	3.19e+06
P62269	40S ribosomal protein S18	RPS18	17.7	358	35	8	8	2.62e+08
P63104	14-3-3 protein zeta/delta	YWHAZ	27.7	333	30	4	7	2.91e+07
P28799	Progranulin	GRN	63.5	321	15	8	8	1.08e+07
P68104	Elongation factor 1-alpha 1	EEF1A1	50.1	316	21	8	8	4.98e+07
Q99623	Prohibitin-2	PHB2	33.3	312	28	8	8	3.15e+07
P07355	Annexin A2	ANXA2	38.6	300	31	9	9	3.37e+07
P09211	Glutathione S-transferase P	GSTP1	23.3	299	52	8	8	7.09e+08
Q71U36	Tubulin alpha-1A chain	TUBA1A	50.1	293	18	7	7	2.99e+07
P22061	Protein-L-isoaspartate (D-aspartate) O-methyltransferase	PCMT1	24.6	264	24	5	5	4.37e+07
P0DMV8	Heat shock 70 kDa protein 1A	HSPA1A	70	263	15	6	8	4.25e+06
P04406	Glyceraldehyde-3-phosphate dehydrogenase	GAPDH	36	256	23	6	6	3.70e+07
P04075	Fructose-bisphosphate aldolase A	ALDOA	39.4	249	18	6	6	1.70e+07
P30101	Protein disulfide-isomerase A3	PDIA3	56.7	244	15	7	7	7.99e+06
P01834	Immunoglobulin kappa constant	IGKC	11.8	243	35	2	2	7.39e+06
P31946	14-3-3 protein beta/alpha	YWHAB	28.1	241	23	1	6	8.64e+06
P61247	40S ribosomal protein S3a	RPS3A	29.9	234	21	6	6	2.06e+07
P14618	Pyruvate kinase PKM	PKM	57.9	216	13	7	7	1.54e+07
P62805	Histone H4	H4C1	11.4	213	39	4	4	7.30e+07
P06454	Prothymosin alpha	PTMA	12.2	213	14	3	3	6.00e+06
P62258	14-3-3 protein epsilon	YWHAE	29.2	211	19	3	5	4.24e+06
P06733	Alpha-enolase	ENO1	47.1	208	8	5	5	2.72e+07
P35232	Prohibitin	PHB	29.8	191	15	5	5	2.32e+07

P27348	14-3-3 protein theta	YWHAQ	27.7	181	19	1	5	
P13639	Elongation factor 2	EEF2	95.3	175	7	6	6	1.51e+07
Q5QNW6	Histone H2B type 2-F	H2BC18	13.9	174	24	1	3	2.15e+06
P01040	Cystatin-A	CSTA	11	169	60	5	5	1.74e+07
Q16778	Histone H2B type 2-E	H2BC21	13.9	166	23	1	3	3.56e+07
P39019	40S ribosomal protein S19	RPS19	16.1	165	28	5	5	4.92e+07
P09651	Heterogeneous nuclear ribonucleoprotein A1	HNRNPA1	38.7	164	14	4	5	8.95e+06
P29401	Transketolase	TKT	67.8	162	9	5	5	6.48e+06
P06748	Nucleophosmin	NPM1	32.6	159	11	3	3	1.48e+07
P62263	40S ribosomal protein S14	RPS14	16.3	158	30	3	3	7.65e+06
P67809	Y-box-binding protein 1	YBX1	35.9	158	17	3	3	2.19e+06
P14923	Junction plakoglobin	JUP	81.7	151	6	4	4	3.79e+06
P13667	Protein disulfide-isomerase A4	PDIA4	72.9	146	7	4	4	4.89e+06
P23528	Cofilin-1	CFL1	18.5	146	24	4	4	4.71e+06
P67936	Tropomyosin alpha-4 chain	TPM4	28.5	145	17	5	5	1.01e+07
Q06830	Peroxiredoxin-1	PRDX1	22.1	144	24	5	5	2.05e+07
P60174	Triosephosphate isomerase	TPI1	26.7	142	13	3	3	8.99e+06
P31943	Heterogeneous nuclear ribonucleoprotein H	HNRNPH1	49.2	140	8	2	4	8.41e+06
Q08554	Desmocollin-1	DSC1	99.9	139	4	3	3	8.26e+06
P61981	14-3-3 protein gamma	YWHAG	28.3	138	17	1	5	5.41e+05
P81605	Dermcidin	DCD	11.3	137	23	3	3	4.80e+07
P19338	Nucleolin	NCL	76.6	135	7	5	5	1.67e+06
P07237	Protein disulfide-isomerase	P4HB	57.1	133	8	3	3	2.19e+06
P20700	Lamin-B1	LMNB1	66.4	131	5	2	4	2.22e+06
Q02413	Desmoglein-1	DSG1	113.7	127	6	5	5	7.43e+06
P52597	Heterogeneous nuclear ribonucleoprotein F	HNRNPF	45.6	126	11	2	4	2.58e+06
Q6UWP8	Suprabasin	SBSN	60.5	126	9	2	2	7.35e+05
P14625	Endoplasmin	HSP90B1	92.4	123	5	3	4	3.30e+06
P35268	60S ribosomal protein L22	RPL22	14.8	118	23	3	3	8.35e+06
Q04917	14-3-3 protein eta	YWHAH	28.2	117	15	1	4	5.03e+05
P22626	Heterogeneous nuclear ribonucleoproteins A2/B1	HNRNPA2B1	37.4	116	15	4	5	5.49e+06
P46782	40S ribosomal protein S5	RPS5	22.9	115	14	4	4	1.72e+07
P23396	40S ribosomal protein S3	RPS3	26.7	114	12	3	3	3.38e+06
P21333	Filamin-A	FLNA	280.6	112	1	3	3	2.33e+06
P52272	Heterogeneous nuclear ribonucleoprotein M	HNRNPM	77.5	110	6	4	4	3.08e+06
P62249	40S ribosomal protein S16	RPS16	16.4	109	19	3	3	5.78e+06
P84090	Enhancer of rudimentary homolog	ERH	12.3	99	27	3	3	3.06e+06
P37802	Transgelin-2	TAGLN2	22.4	97	10	2	2	4.85e+06
P13929	Beta-enolase	ENO3	47	97	3	1	1	2.18e+06
P62987	Ubiquitin-60S ribosomal protein L40	UBA52	14.7	96	20	2	2	6.97e+06
P62826	GTP-binding nuclear protein Ran	RAN	24.4	95	13	3	3	4.04e+06
O00571	ATP-dependent RNA helicase DDX3X	DDX3X	73.2	95	5	3	3	2.57e+06
Q03252	Lamin-B2	LMNB2	69.9	95	4	1	3	4.70e+05
P08708	40S ribosomal protein S17	RPS17	15.5	94	7	1	1	1.07e+07
P01857	Immunoglobulin heavy constant gamma 1	IGHG1	36.1	93	8	2	2	1.92e+06
Q01105	Protein SET	SET	33.5	91	7	2	2	3.64e+06
P62280	40S ribosomal protein S11	RPS11	18.4	90	15	3	3	1.31e+07
Q5D862	Filaggrin-2	FLG2	247.9	88	1	2	2	1.64e+06
P53611	Geranylgeranyl transferase type-2 subunit beta	RABGGTB	36.9	87	6	2	2	5.52e+06
Q9NZT1	Calmodulin-like protein 5	CALML5	15.9	86	10	2	2	5.27e+06
P23526	Adenosylhomocysteinase	AHCY	47.7	84	6	2	2	
P30050	60S ribosomal protein L12	RPL12	17.8	82	15	2	2	3.40e+06
P26641	Elongation factor 1-gamma	EEF1G	50.1	81	5	3	3	1.81e+06
O75955	Flotillin-1	FLOT1	47.3	81	7	2	2	1.44e+06
Q16777	Histone H2A type 2-C	H2AC20	14	80	12	1	2	2.96e+07
P11940	Polyadenylate-binding protein 1	PABPC1	70.6	80	4	3	3	4.12e+06
P84243	Histone H3.3	H3-3A	15.3	80	10	2	2	1.60e+07

P05455	Lupus La protein	SSB	46.8	80	3	1	1	
P10599	Thioredoxin	TXN	11.7	78	12	1	1	5.65e+06
P08574	Cytochrome c1, heme protein, mitochondrial	CYC1	35.4	77	7	2	2	1.53e+06
P12273	Prolactin-inducible protein	PIP	16.6	76	13	2	2	3.73e+06
P62277	40S ribosomal protein S13	RPS13	17.2	76	13	2	2	9.87e+06
P50990	T-complex protein 1 subunit theta	CCT8	59.6	74	6	3	3	5.18e+06
P15311	Ezrin	EZR	69.4	73	3	2	2	2.92e+06
P0DOY2	Immunoglobulin lambda constant 2	IGLC2	11.3	73	24	1	2	5.30e+06
P60842	Eukaryotic initiation factor 4A-I	EIF4A1	46.1	72	5	2	2	4.75e+06
P01876	Immunoglobulin heavy constant alpha 1	IGHA1	37.6	72	5	2	2	3.66e+06
P0DP25	Calmodulin-3	CALM3	16.8	71	12	3	3	7.83e+06
P08865	40S ribosomal protein SA	RPSA	32.8	71	7	2	2	2.19e+06
P00338	L-lactate dehydrogenase A chain	LDHA	36.7	70	6	2	2	5.51e+06
P05109	Protein S100-A8	S100A8	10.8	69	20	2	2	4.03e+06
P31025	Lipocalin-1	LCN1	19.2	68	6	1	1	1.32e+06
P46783	40S ribosomal protein S10	RPS10	18.9	67	8	2	2	1.00e+07
P07737	Profilin-1	PFN1	15	67	10	1	1	3.26e+06
P49368	T-complex protein 1 subunit gamma	CCT3	60.5	65	3	2	2	1.61e+06
P31942	Heterogeneous nuclear ribonucleoprotein H3	HNRNPH3	36.9	64	6	1	2	
P06702	Protein S100-A9	S100A9	13.2	64	25	2	2	2.80e+06
P61604	10 kDa heat shock protein, mitochondrial	HSPE1	10.9	63	14	1	1	
P69905	Hemoglobin subunit alpha	HBA1	15.2	63	15	2	2	5.31e+06
P05388	60S acidic ribosomal protein P0	RPLP0	34.3	63	6	2	2	5.06e+06
Q8IUE6	Histone H2A type 2-B	H2AC21	14	61	12	1	2	2.39e+07
P27797	Calreticulin	CALR	48.1	61	2	1	1	2.58e+06
P50991	T-complex protein 1 subunit delta	CCT4	57.9	61	3	2	2	2.54e+06
P25705	ATP synthase subunit alpha, mitochondrial	ATP5F1A	59.7	60	3	2	2	4.28e+06
P07195	L-lactate dehydrogenase B chain	LDHB	36.6	60	7	2	2	4.08e+06
Q01469	Fatty acid-binding protein 5	FABP5	15.2	59	7	1	1	4.07e+06
Q08380	Galectin-3-binding protein	LGALS3BP	65.3	59	2	1	1	7.06e+05
P04279	Semenogelin-1	SEMG1	52.1	59	8	2	2	
P20930	Filaggrin	FLG	434.9	59	2	2	2	4.75e+06
P27824	Calnexin	CANX	67.5	58	3	2	2	1.03e+06
O75874	Isocitrate dehydrogenase [NADP] cytoplasmic	IDH1	46.6	58	3	1	1	7.87e+05
P49411	Elongation factor Tu, mitochondrial	TUFM	49.5	58	4	2	2	3.02e+06
P31944	Caspase-14	CASP14	27.7	57	8	2	2	6.92e+06
P24844	Myosin regulatory light polypeptide 9	MYL9	19.8	56	5	1	1	1.47e+06
P62829	60S ribosomal protein L23	RPL23	14.9	56	18	2	2	3.54e+06
Q93070	Ecto-ADP-ribosyltransferase 4	ART4	35.9	56	2	1	1	1.60e+07
P12236	ADP/ATP translocase 3	SLC25A6	32.8	56	6	2	2	1.37e+07
P26599	Polypyrimidine tract-binding protein 1	PTBP1	57.2	55	2	1	1	1.38e+06
P51572	B-cell receptor-associated protein 31	BCAP31	28	55	3	1	1	
Q13409	Cytoplasmic dynein 1 intermediate chain 2	DYNC1I2	71.4	55	2	1	1	
B9A064	Immunoglobulin lambda-like polypeptide 5	IGLL5	23	55	11	1	2	1.75e+06
P17844	Probable ATP-dependent RNA helicase DDX5	DDX5	69.1	54	3	2	2	8.00e+05
P78371	T-complex protein 1 subunit beta	CCT2	57.5	54	2	1	1	9.86e+05
P26373	60S ribosomal protein L13	RPL13	24.2	53	4	1	1	
P48643	T-complex protein 1 subunit epsilon	CCT5	59.6	52	2	1	1	1.22e+06
Q07020	60S ribosomal protein L18	RPL18	21.6	52	7	1	1	
P39687	Acidic leucine-rich nuclear	ANP32A	28.6	52	5	1	1	1.24e+05

	phosphoprotein 32 family member A							
P13010	X-ray repair cross-complementing protein 5	XRCC5	82.7	51	2	1	1	
P02765	Alpha-2-HS-glycoprotein	AHSG	39.3	51	3	1	1	
P16402	Histone H1.3	H1-3	22.3	51	5	1	1	2.30e+06
P62913	60S ribosomal protein L11	RPL11	20.2	50	9	2	2	4.31e+06
P59665	Neutrophil defensin 1	DEFA1	10.2	50	10	1	1	1.66e+06
P59190	Ras-related protein Rab-15	RAB15	24.4	49	5	1	1	1.38e+06
P61978	Heterogeneous nuclear ribonucleoprotein K	HNRNPK	50.9	49	4	1	1	9.89e+05
O15258	Protein RER1	RER1	22.9	49	7	1	1	6.94e+05
Q99832	T-complex protein 1 subunit eta	CCT7	59.3	49	2	1	1	1.39e+06
P35637	RNA-binding protein FUS	FUS	53.4	48	7	2	2	
P12814	Alpha-actinin-1	ACTN1	103	46	1	1	1	9.02e+05
Q6ZVX7	F-box only protein 50	NCCRP1	30.8	45	4	1	1	
Q13765	Nascent polypeptide-associated complex subunit alpha	NACA	23.4	44	7	1	1	5.85e+05
Q99497	Parkinson disease protein 7	PARK7	19.9	44	4	1	1	1.03e+06
P29508	Serpin B3	SERPINB3	44.5	44	3	1	1	1.35e+06
Q00688	Peptidyl-prolyl cis-trans isomerase FKBP3	FKBP3	25.2	44	5	1	1	8.01e+05
P09622	Dihydrolipoyl dehydrogenase, mitochondrial	DLD	54.1	43	2	1	1	7.63e+05
P18669	Phosphoglycerate mutase 1	PGAM1	28.8	43	11	2	2	9.17e+06
P08579	U2 small nuclear ribonucleoprotein B''	SNRPB2	25.5	42	5	1	1	
P60866	40S ribosomal protein S20	RPS20	13.4	41	9	1	1	3.06e+06
Q99729	Heterogeneous nuclear ribonucleoprotein A/B	HNRNPAB	36.2	41	4	1	1	
P62081	40S ribosomal protein S7	RPS7	22.1	41	4	1	1	3.32e+06
Q9NQH7	Xaa-Pro aminopeptidase 3	XPNPEP3	57	40	1	1	1	
P14174	Macrophage migration inhibitory factor	MIF	12.5	40	10	1	1	
P28072	Proteasome subunit beta type-6	PSMB6	25.3	40	4	1	1	8.25e+05
Q08J23	RNA cytosine C (5)-methyltransferase NSUN2	NSUN2	86.4	39	1	1	1	1.18e+06
Q15233	Non-POU domain-containing octamer-binding protein	NONO	54.2	39	2	1	1	1.44e+06
Q15517	Corneodesmosin	CDSN	51.5	39	3	1	1	
P60660	Myosin light polypeptide 6	MYL6	16.9	39	6	1	1	1.66e+06
Q00325	Phosphate carrier protein, mitochondrial	SLC25A3	40.1	39	3	1	1	3.13e+06
Q14254	Flotillin-2	FLOT2	47	39	3	1	1	
Q9H853	Putative tubulin-like protein alpha-4B	TUBA4B	27.5	38	5	1	1	
O60506	Heterogeneous nuclear ribonucleoprotein Q	SYNCRIP	69.6	38	2	1	1	
P68871	Hemoglobin subunit beta	HBB	16	38	9	1	1	
Q15365	Poly (rC)-binding protein 1	PCBP1	37.5	38	3	1	1	9.61e+05
P62273	40S ribosomal protein S29	RPS29	6.7	38	20	1	1	3.61e+06
Q07812	Apoptosis regulator BAX	BAX	21.2	38	6	1	1	
Q96N66	Lysophospholipid acyltransferase 7	MBOAT7	52.7	37	3	1	1	
P62906	60S ribosomal protein L10a	RPL10A	24.8	37	4	1	1	3.02e+06
P62244	40S ribosomal protein S15a	RPS15A	14.8	37	5	1	1	1.37e+06
Q92688	Acidic leucine-rich nuclear phosphoprotein 32 family member B	ANP32B	28.8	36	3	1	1	2.07e+06
P29966	Myristoylated alanine-rich C-kinase substrate	MARCKS	31.5	36	5	1	1	1.22e+06
P52907	F-actin-capping protein subunit alpha-1	CAPZA1	32.9	36	5	1	1	
O75122	CLIP-associating protein 2	CLASP2	141	36	1	1	1	
Q8NC51	Plasminogen activator	SERBP1	44.9	36	3	1	1	

	inhibitor 1 RNA-binding protein							
P28074	Proteasome subunit beta type-5	PSMB5	28.5	36	5	1	1	
P30043	Flavin reductase (NADPH)	BLVRB	22.1	36	5	1	1	
P01859	Immunoglobulin heavy constant gamma 2	IGHG2	35.9	35	2	1	1	
Q17RG1	BTB/POZ domain-containing protein KCTD19	KCTD19	104.9	35	1	1	1	2.02e+06
P01036	Cystatin-S	CST4	16.2	35	6	1	1	8.64e+05
P62266	40S ribosomal protein S23	RPS23	15.8	34	8	1	1	1.17e+06
P49006	MARCKS-related protein	MARCKSL1	19.5	33	7	1	1	
O15427	Monocarboxylate transporter 4	SLC16A3	49.4	33	3	1	1	
P25311	Zinc-alpha-2-glycoprotein	AZGP1	34.2	33	3	1	1	
P06744	Glucose-6-phosphate isomerase	GPI	63.1	33	3	1	1	
P31949	Protein S100-A11	S100A11	11.7	33	9	1	1	2.59e+06
P28066	Proteasome subunit alpha type-5	PSMA5	26.4	32	4	1	1	9.23e+05
Q9H4K7	Mitochondrial ribosome-associated GTPase 2	MTG2	43.9	32	2	1	1	
P40939	Trifunctional enzyme subunit alpha, mitochondrial	HADHA	82.9	32	1	1	1	
P40227	T-complex protein 1 subunit zeta	CCT6A	58	31	1	1	1	6.15e+05
Q9ULJ3	Zinc finger and BTB domain-containing protein 21	ZBTB21	118.8	31	1	1	1	
Q86U42	Polyadenylate-binding protein 2	PABPN1	32.7	31	3	1	1	
P61513	60S ribosomal protein L37a	RPL37A	10.3	31	10	1	1	1.28e+06
P46779	60S ribosomal protein L28	RPL28	15.7	31	8	1	1	1.50e+06
P61626	Lysozyme C	LYZ	16.5	30	5	1	1	4.02e+06
P35998	26S proteasome regulatory subunit 7	PSMC2	48.6	30	2	1	1	5.36e+05
P08183	ATP-dependent translocase ABCB1	ABCB1	141.4	30	1	1	1	9.01e+06
Q8NAB2	Kelch repeat and BTB domain-containing protein 3	KBTBD3	69.8	30	1	1	1	2.78e+06
P15531	Nucleoside diphosphate kinase A	NME1	17.1	30	11	1	1	
Q12905	Interleukin enhancer-binding factor 2	ILF2	43	30	3	1	1	1.28e+06
P31151	Protein S100-A7	S100A7	11.5	30	11	1	1	
P62851	40S ribosomal protein S25	RPS25	13.7	30	7	1	1	1.77e+06
Q2PPJ7	Ral GTPase-activating protein subunit alpha-2	RALGAPA2	210.6	29	0	1	1	1.39e+06
P02810	Salivary acidic proline-rich phosphoprotein 1/2	PRH1	17	29	10	1	1	1.77e+06
P15559	NAD (P)H dehydrogenase [quinone] 1	NQO1	30.8	29	4	1	1	1.28e+06
P55072	Transitional endoplasmic reticulum ATPase	VCP	89.3	28	2	1	1	7.94e+05
P62841	40S ribosomal protein S15	RPS15	17	28	5	1	1	8.20e+05
P04040	Catalase	CAT	59.7	27	2	1	1	
P47897	Glutamine--tRNA ligase	QARS1	87.7	27	1	1	1	1.05e+06
P51858	Hepatoma-derived growth factor	HDGF	26.8	26	4	1	1	
Q08188	Protein-glutamine gamma-glutamyltransferase E	TGM3	76.6	26	2	1	1	1.09e+06
P62136	Serine/threonine-protein phosphatase PP1-alpha catalytic subunit	PPP1CA	37.5	26	3	1	1	
P31948	Stress-induced-phosphoprotein 1	STIP1	62.6	26	2	1	1	6.83e+05
Q15084	Protein disulfide-isomerase A6	PDIA6	48.1	25	2	1	1	
P09848	Lactase-phlorizin hydrolase	LCT	218.5	25	0	1	1	3.49e+06

Table S4. List of the 174 proteins in the PMEPA1-overexpression group identified by mass spectrometry.

Accession	Protein names	Gene names	MW [kDa]	Protein score	Sequence coverage (%)	Unique Peptides	Peptides	Abundances
P02545	Prelamin-A/C	LMNA	74.1	1491	40	24	26	1.20e+08
P11021	Endoplasmic reticulum chaperone BiP	HSPA5	72.3	753	30	15	16	6.57e+07
P60709	Actin, cytoplasmic 1	ACTB	41.7	751	38	4	13	4.34e+08
P11142	Heat shock cognate 71 kDa protein	HSPA8	70.9	632	22	9	12	4.78e+07
P02768	Albumin	ALB	69.3	598	23	15	15	1.12e+09
P68133	Actin, alpha skeletal muscle	ACTA1	42	514	27	1	10	3.29e+06
Q86YZ3	Hornerin	HRNR	282.2	488	12	9	9	3.07e+07
P38646	Stress-70 protein, mitochondrial	HSPA9	73.6	477	22	13	13	3.84e+07
P62269	40S ribosomal protein S18	RPS18	17.7	382	53	12	12	3.70e+08
P62701	40S ribosomal protein S4, X isoform	RPS4X	29.6	315	39	12	12	8.34e+07
P68104	Elongation factor 1-alpha 1	EEF1A1	50.1	294	21	8	8	2.88e+07
P61247	40S ribosomal protein S3a	RPS3A	29.9	279	18	5	5	1.51e+07
P07355	Annexin A2	ANXA2	38.6	279	31	9	9	2.64e+07
P07437	Tubulin beta chain	TUBB	49.6	271	17	3	6	1.56e+07
P62805	Histone H4	H4C1	11.4	269	50	5	5	1.89e+08
P04406	Glyceraldehyde-3-phosphate dehydrogenase	GAPDH	36	262	17	5	5	1.14e+07
P09651	Heterogeneous nuclear ribonucleoprotein A1	HNRNPA1	38.7	254	14	3	5	7.64e+06
P10809	60 kDa heat shock protein, mitochondrial	HSPD1	61	251	12	6	6	1.29e+07
P68371	Tubulin beta-4B chain	TUBB4B	49.8	237	13	2	5	1.67e+06
P52272	Heterogeneous nuclear ribonucleoprotein M	HNRNPM	77.5	228	12	7	7	7.63e+06
P63104	14-3-3 protein zeta/delta	YWHAZ	27.7	224	33	5	8	1.61e+07
P28799	Progranulin	GRN	63.5	217	10	5	5	3.95e+06
P22626	Heterogeneous nuclear ribonucleoproteins A2/B1	HNRNPA2B1	37.4	216	17	5	6	1.29e+07
P01834	Immunoglobulin kappa constant	IGKC	11.8	214	35	2	2	6.82e+06
P06733	Alpha-enolase	ENO1	47.1	206	11	5	6	1.85e+07
P0DMV8	Heat shock 70 kDa protein 1A	HSPA1A	70	198	11	4	6	1.64e+06
P0DPH7	Tubulin alpha-3C chain	TUBA3C	49.9	192	12	5	5	1.51e+07
Q99623	Prohibitin-2	PHB2	33.3	187	21	6	6	1.60e+07
Q5QNW6	Histone H2B type 2-F	H2BC18	13.9	182	24	2	4	6.91e+07
P39019	40S ribosomal protein S19	RPS19	16.1	173	28	5	5	2.72e+07
P67809	Y-box-binding protein 1	YBX1	35.9	169	21	3	3	
P14923	Junction plakoglobin	JUP	81.7	167	7	5	5	9.71e+05
P09211	Glutathione S-transferase P	GSTP1	23.3	165	21	4	4	3.30e+07
Q6UWP8	Suprabasin	SBSN	60.5	162	18	3	3	1.78e+06
P81605	Dermcidin	DCD	11.3	159	23	3	3	3.29e+07
P22061	Protein-L-isoaspartate (D-aspartate) O-methyltransferase	PCMT1	24.6	154	20	4	4	1.52e+07
P35232	Prohibitin	PHB	29.8	147	14	4	4	6.61e+06
P07910	Heterogeneous nuclear ribonucleoproteins C1/C2	HNRNPC	33.7	147	17	6	6	1.55e+08
P06454	Prothymosin alpha	PTMA	12.2	143	13	2	2	1.05e+06
P07900	Heat shock protein HSP 90-alpha	HSP90AA1	84.6	140	6	1	5	
P62987	Ubiquitin-60S ribosomal protein L40	UBA52	14.7	140	31	4	4	2.36e+07
P05109	Protein S100-A8	S100A8	10.8	140	40	4	4	2.45e+07
P27348	14-3-3 protein theta	YWHAQ	27.7	138	16	1	4	
Q16778	Histone H2B type 2-E	H2BC21	13.9	136	23	1	3	1.33e+06
P62263	40S ribosomal protein S14	RPS14	16.3	133	30	3	3	8.98e+06
P0DOX8	Immunoglobulin lambda-	--	22.8	127	14	3	4	1.11e+07

	1 light chain							
P08238	Heat shock protein HSP 90-beta	HSP90AB1	83.2	125	6	1	5	8.78e+06
Q5D862	Filaggrin-2	FLG2	247.9	123	4	4	4	1.96e+06
P60660	Myosin light polypeptide 6	MYL6	16.9	121	15	2	2	4.65e+06
P10599	Thioredoxin	TXN	11.7	120	21	2	2	9.27e+06
P04075	Fructose-bisphosphate aldolase A	ALDOA	39.4	119	10	4	4	6.92e+06
P14618	Pyruvate kinase PKM	PKM	57.9	116	9	5	5	5.73e+06
P06748	Nucleophosmin	NPM1	32.6	116	11	3	3	7.95e+06
P31943	Heterogeneous nuclear ribonucleoprotein H	HNRNPH1	49.2	115	7	2	3	5.24e+06
P35268	60S ribosomal protein L22	RPL22	14.8	115	23	3	3	6.76e+06
P62249	40S ribosomal protein S16	RPS16	16.4	114	18	3	3	1.14e+07
P06702	Protein S100-A9	S100A9	13.2	111	31	3	3	9.72e+06
P01040	Cystatin-A	CSTA	11	111	40	4	4	1.29e+07
P31946	14-3-3 protein beta/alpha	YWHAB	28.1	101	15	1	4	
P20700	Lamin-B1	LMNB1	66.4	100	3	1	3	
P62280	40S ribosomal protein S11	RPS11	18.4	99	15	3	3	1.27e+07
Q71UI9	Histone H2A.V	H2AZ2	13.5	96	23	2	3	1.08e+08
P62244	40S ribosomal protein S15a	RPS15A	14.8	94	22	3	3	2.67e+06
P08708	40S ribosomal protein S17	RPS17	15.5	91	7	1	1	7.22e+06
P84090	Enhancer of rudimentary homolog	ERH	12.3	91	27	3	3	2.49e+06
P30101	Protein disulfide-isomerase A3	PDIA3	56.7	88	6	3	3	2.62e+06
Q08554	Desmocollin-1	DSC1	99.9	87	4	3	3	5.71e+06
Q03252	Lamin-B2	LMNB2	69.9	87	4	1	3	
P51991	Heterogeneous nuclear ribonucleoprotein A3	HNRNPA3	39.6	87	4	1	2	
P62829	60S ribosomal protein L23	RPL23	14.9	86	18	2	2	2.82e+06
P69905	Hemoglobin subunit alpha	HBA1	15.2	85	15	2	2	4.41e+06
P84243	Histone H3.3	H3-3A	15.3	83	10	2	2	3.08e+07
Q8IUE6	Histone H2A type 2-B	H2AC21	14	80	12	1	2	3.42e+06
P0DOY2	Immunoglobulin lambda constant 2	IGLC2	11.3	78	24	1	2	3.05e+06
P08574	Cytochrome c1, heme protein, mitochondrial	CYC1	35.4	75	7	2	2	
P46782	40S ribosomal protein S5	RPS5	22.9	75	14	4	4	8.47e+06
P15924	Desmoplakin	DSP	331.6	74	1	3	3	9.04e+05
P31944	Caspase-14	CASP14	27.7	73	12	3	3	6.39e+06
P60866	40S ribosomal protein S20	RPS20	13.4	72	19	2	2	4.14e+06
P26373	60S ribosomal protein L13	RPL13	24.2	72	9	2	2	9.79e+05
P37802	Transgelin-2	TAGLN2	22.4	71	10	2	2	1.95e+06
P31942	Heterogeneous nuclear ribonucleoprotein H3	HNRNPH3	36.9	70	6	1	2	
P0DP25	Calmodulin-3	CALM3	16.8	69	11	2	2	4.64e+06
P62277	40S ribosomal protein S13	RPS13	17.2	68	13	2	2	9.29e+06
P13929	Beta-enolase	ENO3	47	67	5	1	2	
P67936	Tropomyosin alpha-4 chain	TPM4	28.5	66	7	2	2	3.58e+06
Q9UKM9	RNA-binding protein Raly	RALY	32.4	64	6	2	2	2.54e+06
P31947	14-3-3 protein sigma	SFN	27.8	64	12	1	3	3.81e+05
Q9NZT1	Calmodulin-like protein 5	CALML5	15.9	63	10	2	2	5.43e+06
P26599	Polypyrimidine tract-binding protein 1	PTBP1	57.2	63	4	2	2	1.67e+06
P46783	40S ribosomal protein S10	RPS10	18.9	61	8	2	2	5.97e+06
P20930	Filaggrin	FLG	434.9	61	2	2	2	3.57e+06
Q06830	Peroxiredoxin-1	PRDX1	22.1	60	9	2	2	4.30e+06
Q01105	Protein SET	SET	33.5	59	4	1	1	8.60e+05
P01857	Immunoglobulin heavy constant gamma 1	IGHG1	36.1	58	6	1	2	
P00338	L-lactate dehydrogenase	LDHA	36.7	58	6	2	2	2.76e+06

	A chain							
P12273	Prolactin-inducible protein	PIP	16.6	57	8	1	1	2.17e+06
P16402	Histone H1.3	H1-3	22.3	56	5	1	1	1.07e+06
O75874	Isocitrate dehydrogenase [NADP] cytoplasmic	IDH1	46.6	54	3	1	1	
P08865	40S ribosomal protein SA	RPSA	32.8	53	4	1	1	1.22e+06
P59665	Neutrophil defensin 1	DEFA1	10.2	52	10	1	1	3.57e+06
P07339	Cathepsin D	CTSD	44.5	52	4	2	2	2.53e+06
P29508	Serpin B3	SERPINB3	44.5	52	4	2	2	1.01e+06
Q00325	Phosphate carrier protein, mitochondrial	SLC25A3	40.1	50	3	1	1	2.82e+06
P62306	Small nuclear ribonucleoprotein F	SNRPF	9.7	49	15	1	1	
O00571	ATP-dependent RNA helicase DDX3X	DDX3X	73.2	49	2	1	1	
P30050	60S ribosomal protein L12	RPL12	17.8	49	5	1	1	2.15e+06
P62266	40S ribosomal protein S23	RPS23	15.8	48	16	2	2	2.51e+06
P02765	Alpha-2-HS-glycoprotein	AHSG	39.3	45	3	1	1	
P62913	60S ribosomal protein L11	RPL11	20.2	45	4	1	1	1.83e+06
P01859	Immunoglobulin heavy constant gamma 2	IGHG2	35.9	45	5	1	2	5.36e+08
P51572	B-cell receptor-associated protein 31	BCAP31	28	45	3	1	1	
P01876	Immunoglobulin heavy constant alpha 1	IGHA1	37.6	45	3	1	1	8.88e+05
P62906	60S ribosomal protein L10a	RPL10A	24.8	45	4	1	1	2.54e+06
P23396	40S ribosomal protein S3	RPS3	26.7	45	3	1	1	1.62e+06
P68871	Hemoglobin subunit beta	HBB	16	44	9	1	1	
P07237	Protein disulfide-isomerase	P4HB	57.1	44	3	1	1	
P05455	Lupus La protein	SSB	46.8	44	3	1	1	
O75152	Zinc finger CCCH domain-containing protein 11A	ZC3H11A	89.1	43	1	1	1	1.94e+08
P62081	40S ribosomal protein S7	RPS7	22.1	43	4	1	1	1.70e+06
Q17RG1	BTB/POZ domain-containing protein KCTD19	KCTD19	104.9	42	1	1	1	1.50e+08
Q6ZVX7	F-box only protein 50	NCCRP1	30.8	42	4	1	1	
P53804	E3 ubiquitin-protein ligase TTC3 OS=Homo sapiens OX=9606 GN=TTC3 PE=1 SV=2	TTC3	229.87	42	6	1	1	
P62273	40S ribosomal protein S29	RPS29	6.7	42	20	1	1	3.41e+06
P25705	ATP synthase subunit alpha, mitochondrial	ATP5F1A	59.7	41	1	1	1	1.65e+06
P06744	Glucose-6-phosphate isomerase	GPI	63.1	41	3	1	1	
P23528	Cofilin-1	CFL1	18.5	41	7	1	1	1.46e+06
P61604	10 kDa heat shock protein, mitochondrial	HSPE1	10.9	41	14	1	1	
Q9H2G2	STE20-like serine/threonine-protein kinase	SLK	142.6	40	1	1	1	1.79e+07
Q96DR8	Mucin-like protein 1	MUCL1	9	40	10	1	1	1.75e+06
P31025	Lipocalin-1	LCN1	19.2	40	6	1	1	7.55e+05
P35637	RNA-binding protein FUS	FUS	53.4	40	3	1	1	9.59e+05
P13639	Elongation factor 2	EEF2	95.3	39	1	1	1	
P29401	Transketolase	TKT	67.8	39	1	1	1	1.89e+06
P62826	GTP-binding nuclear protein Ran	RAN	24.4	39	4	1	1	2.05e+06
P05388	60S acidic ribosomal protein P0	RPLP0	34.3	37	3	1	1	1.17e+06
O75122	CLIP-associating protein 2	CLASP2	141	36	1	1	1	1.91e+06
P23526	Adenosylhomocysteinase	AHCY	47.7	36	3	1	1	
Q08380	Galectin-3-binding protein	LGALS3BP	65.3	35	2	1	1	
Q13185	Chromobox protein	CBX3	20.8	35	6	1	1	

	homolog 3							
P08579	U2 small nuclear ribonucleoprotein B''	SNRPB2	25.5	34	5	1	1	
P61513	60S ribosomal protein L37a	RPL37A	10.3	34	10	1	1	1.68e+06
P19338	Nucleolin	NCL	76.6	33	1	1	1	
Q9UGC7	Peptide chain release factor 1-like, mitochondrial	MTRF1L	43.6	33	2	1	1	4.83e+06
P60174	Triosephosphate isomerase	TPI1	26.7	33	5	1	1	
P26641	Elongation factor 1-gamma	EEF1G	50.1	33	3	1	1	
P42766	60S ribosomal protein L35	RPL35	14.5	33	8	1	1	1.93e+06
Q01469	Fatty acid-binding protein 5	FABP5	15.2	33	7	1	1	2.24e+06
Q12905	Interleukin enhancer-binding factor 2	ILF2	43	32	3	1	1	1.17e+06
Q8TD57	Dynein axonemal heavy chain 3	DNAH3	470.5	32	0	1	1	7.57e+06
Q15517	Corneodesmosin	CDSN	51.5	31	3	1	1	
P49207	60S ribosomal protein L34	RPL34	13.3	31	6	1	1	9.77e+05
P31949	Protein S100-A11	S100A11	11.7	31	9	1	1	1.48e+06
P61626	Lysozyme C	LYZ	16.5	31	5	1	1	2.22e+06
A8MX80	Putative UPF0607 protein ENSP00000383144	--	37.6	30	2	1	1	3.46e+06
Q2PPJ7	Ral GTPase-activating protein subunit alpha-2	RALGAPA2	210.6	29	0	1	1	1.23e+06
P30043	Flavin reductase (NADPH)	BLVRB	22.1	29	5	1	1	1.16e+06
Q02413	Desmoglein-1	DSG1	113.7	29	1	1	1	
P49411	Elongation factor Tu, mitochondrial	TUFM	49.5	29	2	1	1	2.94e+06
Q93070	Ecto-ADP-ribosyltransferase 4	ART4	35.9	29	2	1	1	7.69e+06
Q96HS1	Serine/threonine-protein phosphatase PGAM5, mitochondrial	PGAM5	32	29	4	1	1	5.93e+05
Q14254	Flotillin-2	FLOT2	47	28	3	1	1	
P46781	40S ribosomal protein S9	RPS9	22.6	28	8	1	1	
P15311	Ezrin	EZR	69.4	28	1	1	1	9.56e+05
Q8WVV4	Protein POF1B	POF1B	68	28	2	1	1	
Q8N8Y5	Zinc finger protein 41 homolog	ZFP41	22.8	27	5	1	1	1.75e+08
Q9BX63	Fanconi anemia group J protein	BRIP1	140.8	27	1	1	1	7.13e+05
P15531	Nucleoside diphosphate kinase A	NME1	17.1	26	11	1	1	
P62753	40S ribosomal protein S6	RPS6	28.7	26	4	1	1	1.09e+06
Q9ULD0	2-oxoglutarate dehydrogenase-like, mitochondrial	OGDHL	114.4	25	1	1	1	7.88e+06
P37108	Signal recognition particle 14 kDa protein	SRP14	14.6	24	6	1	1	5.88e+05
P09848	Lactase-phlorizin hydrolase	LCT	218.5	24	0	1	1	2.01e+06
P15880	40S ribosomal protein S2	RPS2	31.3	23	4	1	1	
P27797	Calreticulin	CALR	48.1	21	2	1	1	

The rows in red represent the key proteins 14-3-3σ and TTC3 identified in this study through mass spectrometry.

Table S5. The 30 proteins uniquely interacted with PMEPA1-overexpression group in mass spectrometry analysis.

Accession	Protein names	Gene names
Q96HS1	Serine/threonine-protein phosphatase PGAM5, mitochondrial (EC 3.1.3.16) (Bcl-XL-binding protein v68) (Phosphoglycerate mutase family member 5)	PGAM5
Q9UKM9	RNA-binding protein Raly (Autoantigen p542) (Heterogeneous nuclear ribonucleoprotein C-like 2) (hnRNP core protein C-like 2) (hnRNP associated with lethal yellow protein homolog)	RALY HNRPCL2 P542
O75152	Zinc finger CCCH domain-containing protein 11A	ZC3H11A KIAA0663 ZC3HDC11A
P07339	Cathepsin D (EC 3.4.23.5) [Cleaved into: Cathepsin D light chain; Cathepsin D heavy chain]	CTSD CPSD
Q71UI9	Histone H2A.V (H2A.F/Z) (H2A.Z variant histone 2)	H2AZ2 H2AFV H2AV
P0DOX8	Immunoglobulin lambda-1 light chain (Immunoglobulin lambda-1 light chain MCG)	
Q9ULD0	2-oxoglutarate dehydrogenase-like, mitochondrial (EC 1.2.4.2) (2-oxoglutarate dehydrogenase complex component E1-like) (OGDC-E1-like) (Alpha-ketoglutarate dehydrogenase-like)	OGDHL KIAA1290
Q13185	Chromobox protein homolog 3 (HECH) (Heterochromatin protein 1 homolog gamma) (HP1 gamma) (Modifier 2 protein)	CBX3
P62306	Small nuclear ribonucleoprotein F (snRNP-F) (Sm protein F) (Sm-F) (SmF)	SNRPF PBSCF
P07910	Heterogeneous nuclear ribonucleoproteins C1/C2 (hnRNP C1/C2)	HNRNPC HNRPC
P37108	Signal recognition particle 14 kDa protein (SRP14) (18 kDa Alu RNA-binding protein)	SRP14
P62753	Small ribosomal subunit protein eS6 (40S ribosomal protein S6) (Phosphoprotein NP33)	RPS6 OK/SW-cl.2
P42766	Large ribosomal subunit protein uL29 (60S ribosomal protein L35)	RPL35
Q9H2G2	STE20-like serine/threonine-protein kinase (STE20-like kinase) (hSLK) (EC 2.7.11.1) (CTCL tumor antigen se20-9) (STE20-related serine/threonine-protein kinase) (STE20-related kinase) (Serine/threonine-protein kinase 2)	SLK KIAA0204 STK2
Q8N8Y5	Zinc finger protein 41 homolog (Zfp-41)	ZFP41
A8MX80	Putative UPF0607 protein ENSP00000383144	
P51991	Heterogeneous nuclear ribonucleoprotein A3 (hnRNP A3)	HNRNPA3 HNRPA3
P68133	Actin, alpha skeletal muscle (EC 3.6.4.-) (Alpha-actin-1) [Cleaved into: Actin, alpha skeletal muscle, intermediate form]	ACTA1 ACTA
Q8TD57	Dynein axonemal heavy chain 3 (Axonemal beta dynein heavy chain 3) (HsADHC3) (Ciliary dynein heavy chain 3) (Dnahc3-b)	DNAH3 DNAHC3B
Q9BX63	Fanconi anemia group J protein (Protein FACJ) (EC 3.6.4.13) (ATP-dependent RNA helicase BRIP1) (BRCA1-associated C-terminal helicase 1) (BRCA1-interacting protein C-terminal helicase 1) (BRCA1-interacting protein 1)	BRIP1 BACH1 FANCJ
P31947	14-3-3 protein sigma (Epithelial cell marker protein 1) (Stratifin)	SFN HME1
P53804	E3 ubiquitin-protein ligase TTC3 OS=Homo sapiens OX=9606 GN=TTC3 PE=1 SV=2	TTC3
Q96DR8	Mucin-like protein 1 (Protein BS106) (Small breast epithelial mucin)	MUCL1 SBEM UNQ590/PRO1160
P0DPH7	Tubulin alpha-3C chain (EC 3.6.5.-) (Alpha-tubulin 2) (Alpha-tubulin 3C) (Tubulin alpha-2 chain) [Cleaved into: Detyrosinated tubulin alpha-3C chain]	TUBA3C TUBA2
Q9UGC7	Peptide chain release factor 1-like, mitochondrial (Mitochondrial translational release factor 1-like) (mtRF1a)	MTRF1L MTRF1A
P49207	Large ribosomal subunit protein eL34 (60S ribosomal protein L34)	RPL34
P46781	Small ribosomal subunit protein uS4 (40S ribosomal protein S9)	RPS9
P15924	Desmoplakin (DP) (250/210 kDa paraneoplastic pemphigus antigen)	DSP
Q8WVV4	Protein POF1B (Premature ovarian failure protein 1B)	POF1B
P15880	Small ribosomal subunit protein uS5 (40S ribosomal protein S2) (40S ribosomal protein S4) (Protein LLRep3)	RPS2 RPS4
